# Impact of the 13-Valent Pneumococcal Conjugate Vaccine on Clinical and Hypoxemic Childhood Pneumonia over Three Years in Central Malawi: An Observational Study

**DOI:** 10.1371/journal.pone.0168209

**Published:** 2017-01-04

**Authors:** Eric D. McCollum, Bejoy Nambiar, Rashid Deula, Beatiwel Zadutsa, Austin Bondo, Carina King, James Beard, Harry Liyaya, Limangeni Mankhambo, Marzia Lazzerini, Charles Makwenda, Gibson Masache, Naor Bar-Zeev, Peter N. Kazembe, Charles Mwansambo, Norman Lufesi, Anthony Costello, Ben Armstrong, Tim Colbourn

**Affiliations:** 1 Institute for Global Health, University College London, London, United Kingdom; 2 Department of Pediatrics, Division of Pulmonology, Johns Hopkins School of Medicine, Baltimore, Maryland, United States of America; 3 Parent and Child Health Initiative Trust, Lilongwe, Malawi; 4 WHO Collaborating Centre for Maternal and Child Health, Institute for Maternal and Child Health IRCCS Burlo Garofolo, Trieste, Italy; 5 Malawi-Liverpool-Wellcome Trust Clinical Research Programme, College of Medicine, University of Malawi, Blantyre, Malawi; 6 Institute of Infection and Global Health, University of Liverpool, Liverpool, United Kingdom; 7 Baylor College of Medicine Children’s Foundation, Lilongwe, Malawi; 8 Ministry of Health, Lilongwe, Malawi; 9 Community Health Sciences Unit, Ministry of Health, Lilongwe, Malawi; 10 Department of Social and Environmental Health Research, London School of Hygiene and Tropical Medicine, London, United Kingdom; Public Health England, UNITED KINGDOM

## Abstract

**Background:**

The pneumococcal conjugate vaccine’s (PCV) impact on childhood pneumonia during programmatic conditions in Africa is poorly understood. Following PCV13 introduction in Malawi in November 2011, we evaluated the case burden and rates of childhood pneumonia.

**Methods and Findings:**

Between January 1, 2012-June 30, 2014 we conducted active pneumonia surveillance in children <5 years at seven hospitals, 18 health centres, and with 38 community health workers in two districts, central Malawi. Eligible children had clinical pneumonia per Malawi guidelines, defined as fast breathing only, chest indrawing +/- fast breathing, or, ≥1 clinical danger sign. Since pulse oximetry was not in the Malawi guidelines, oxygenation <90% defined hypoxemic pneumonia, a distinct category from clinical pneumonia. We quantified the pneumonia case burden and rates in two ways. We compared the period immediately following vaccine introduction (early) to the period with >75% three-dose PCV13 coverage (post). We also used multivariable time-series regression, adjusting for autocorrelation and exploring seasonal variation and alternative model specifications in sensitivity analyses.

The early versus post analysis showed an increase in cases and rates of total, fast breathing, and indrawing pneumonia and a decrease in danger sign and hypoxemic pneumonia, and pneumonia mortality. At 76% three-dose PCV13 coverage, versus 0%, the time-series model showed a non-significant increase in total cases (+47%, 95% CI: -13%, +149%, p = 0.154); fast breathing cases increased 135% (+39%, +297%, p = 0.001), however, hypoxemia fell 47% (-5%, -70%, p = 0.031) and hospital deaths decreased 36% (-1%, -58%, p = 0.047) in children <5 years. We observed a shift towards disease without danger signs, as the proportion of cases with danger signs decreased by 65% (-46%, -77%, p<0.0001). These results were generally robust to plausible alternative model specifications.

**Conclusions:**

Thirty months after PCV13 introduction in Malawi, the health system burden and rates of the severest forms of childhood pneumonia, including hypoxemia and death, have markedly decreased.

## Introduction

Pneumonia is the second most frequent killer of children <5 years old worldwide.[1] Nearly one million children died of pneumonia in 2013, most were in Africa.[1] *Streptococcus pneumoniae* is a major contributor to global disease burden, accounting for ~14 million pneumonia cases and more than 1/3 of pneumonia-associated deaths per 2009 estimates.[2] Children <5 years in Africa are especially vulnerable: rates of overall and pneumonia-associated mortality are the highest worldwide.[2]

In high-income countries like the United States, the 7-valent pneumococcal conjugate vaccine (PCV7) dramatically reduced invasive pneumococcal disease (IPD) and clinical pneumonia in children,[3, 4] and induced widespread herd immunity.[5, 6] Non-PCV7 serotype replacement then emerged and slowed these declines while increasing empyema rates.[7–10] Broader valency vaccines were therefore developed. PCV13 targets an additional six serotypes including serotype 19A, a frequently invasive and drug resistant strain,[11–13] and serotypes one and five that are common in Africa.[14, 15] After only two years in the United States PCV13 further reduced IPD and clinical pneumonia paediatric hospitalizations while reversing escalating empyema rates.[6, 13]

Randomized trials in Africa of PCV9 (includes PCV7 serotypes plus serotypes 1 and 5) demonstrated similar vaccine benefits.[16–18] Subsequently, PCV9, PCV10, and more recently PCV13 have been introduced into several African countries, including PCV13 in Malawi in 2011, at a schedule of six, ten, 14 weeks of age, and no booster dose (i.e., 3+0 schedule). To date PCV13’s impact on childhood clinical pneumonia during programmatic conditions in Africa is not well understood.

By conducting widespread active pneumonia surveillance over 30 months at all health system levels and applying two complementary analytic methods, we sought to address this knowledge gap and determine PCV13’s effect on the burden and incidence of clinical and hypoxemic pneumonia cases in <5 year olds in two districts in central Malawi. Our analysis first compared the period just after the introduction of PCV13 (early) to the period with >75% three-dose PCV13 coverage (post). We then employed multivariable time-series regression techniques over the entire study period. We hypothesized *a priori* that PCV13 would reduce the case burden and rate of childhood pneumonia, especially the most severe cases, under the assumption that pneumococcus is both common and lethal in Malawian children with respiratory disease.

## Methods

### Study design and setting

**Fig 1:**
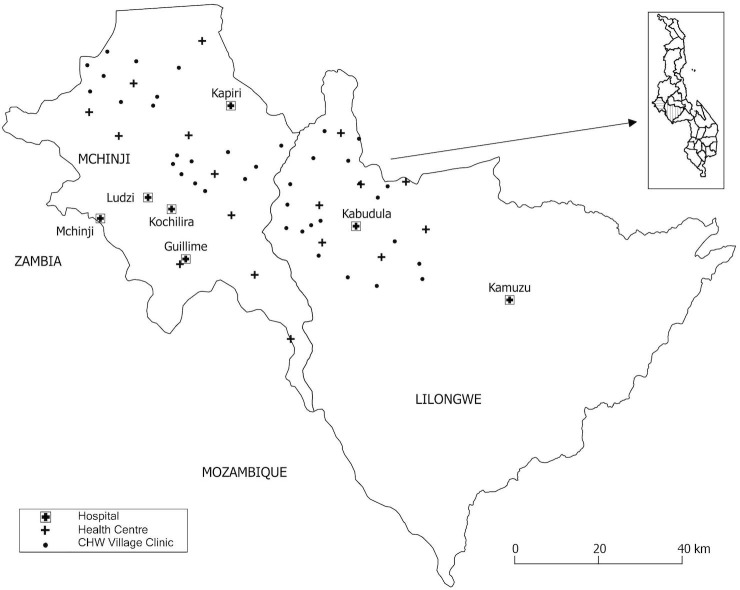
Map of study sites

We conducted prospective, active surveillance, embedded into routine care, between January 1st, 2012-June 30th, 2014 in seven hospitals, 18 outpatient health centres, and by 38 community health workers (CHWs) in Lilongwe and Mchinji district, central Malawi (Fig 1). The catchment population was over 2.3 million people,[19] ~15% of Malawi’s population. Hospitals covered the entire catchment area while health centres covered 28%: Mchinji district, and Kabudula, one health area in Lilongwe district (Fig 1). Hospitals offered limited radiography and oxygen while health centres provided outpatient care without either. Active pneumonia surveillance without pulse oximetry was conducted at hospitals since 2001 as a part of the Child Lung Health Program, but not at health centres or with CHWs.[20] In late 2011 this study introduced pulse oximetry to the hospitals, health centres and CHWs it covers. CHWs covered 3% of the study population (Fig 1). They followed community-based guidelines and prescribed antibiotics, providing care from weekly to bi-weekly held informal village clinics or via home visits.[21]

Individual informed consent was not required since the study collected routine data. The institutional review boards of University College London (protocol 2006/002) and Malawi (protocol 941) provided approval.

### Data collection

Government healthcare staff prospectively collected routine pneumonia data from January 1, 2012. The hospital data form was adapted from a Ministry of Health tool while the health centre and CHW forms were created since no routine data forms previously existed (S1 Appendix). Providers recorded diagnoses and non-invasive peripheral oxygen saturation (SpO_2_) measurements at presentation. SpO_2_ was collected in room air with Masimo® (hospitals) or Lifebox® (health centres and CHWs) oximeters. PCV13 doses were documented from patient-held government-issued health records. The study followed hospitalized patients until hospital outcome, including death. While outpatient follow-up visits are recommended at all levels, they do not routinely occur and so outpatients were excluded from the mortality analysis. Hospitalized patients and outpatients at health centre and CHW level were all included in the analyses of cases of pneumonia. Patients could be counted multiple times during a single illness to allow measurement of PCV13’s effect on medically attended pneumonia episodes (health system pneumonia burden).

We emphasized case ascertainment by integrating trainings and supervision into the Ministry of Health Child Lung Health Program. During the three-month pilot phase from October-December 2011, participating providers attended competency-based trainings of pneumonia guidelines and oximetry use facilitated by a pediatric pulmonologist (EDM). Providers included nurses, non-physician clinicians and CHWs; all passed testing before participating. Those who failed were remediated. Monthly supervision visits occurred at all sites. Supervisors (RD, BZ, HL, LM, EDM) assessed case ascertainment by evaluating provider decision-making with a quiz and observing a patient encounter that included oximetry. Deficiencies were corrected. Supervision also allowed on-the-job training for new providers throughout the study. All staff received retraining in April 2013 facilitated by EDM. All sites met quarterly for data reviews and study progress assessments; hospitals met weekly to conduct quality assurance checks of their surveillance data and, via review of all case records and notes, ensure all patient deaths were recorded.

### Definitions

#### Pneumonia (see Table 1)

We defined pneumonia per Malawi national pneumonia guidelines, previously adapted from World Health Organization (WHO) clinical definitions, and by hypoxemia (SpO_2_<90%).[22]^,^[23] The hypoxemic pneumonia definition overlapped with clinical categories such that patients with hypoxemia also were separately classified into one of the three mutually exclusive clinical pneumonia categories (Table 1).

**Table 1:** Clinical and hypoxemic pneumonia definitions

Pneumonia classification	Definition (signs and symptoms)
**Fast breathing pneumonia**^**1**^	Cough and/or difficulty breathing ***and***
	Fast breathing for age^1^ ***and***
	**No** lower chest indrawing and **no** danger signs^2^
**Chest indrawing pneumonia**	Cough and/or difficulty breathing ***and***
	Lower chest indrawing ***and***
	**No** danger signs^2^
	May or may not have fast breathing for age^1^
**Danger sign pneumonia**	Cough and/or difficulty breathing ***and***
	At least one danger sign^2^
	May or may not have fast breathing for age^1^ or lower chest indrawing
**Hypoxemic pneumonia**	Cough and/or difficulty breathing ***and***
	SpO^2^<90%
	May or may not have fast breathing for age^1^ or lower chest indrawing or danger signs^2^

^1^≥60 breaths/minute if <2 months old, ≥50 breaths/minute 2–11 months old; ≥40 breaths/minute if 12–59 months old

^2^Danger signs are any of the following: central cyanosis (hospital); severe respiratory distress (hospital), stridor in a calm child, inability to drink and/or breastfeed, persistent vomiting, lethargy or unconscious, convulsions), apnea (if 0–2 months of age)

SpO_2_ indicates peripheral oxygen saturation.

#### Early- and post-PCV13

Malawi introduced PCV13 during November-December 2011 in the study area. The vaccine was administered at six, ten, and 14 weeks of age without a booster dose (i.e., 3+0 schedule). If children missed the first dose they could still receive subsequent doses, and a passive catch-up campaign was conducted during the first year of vaccine introduction for children older than 14 weeks but younger than one year of age at time of first dose. We compared the period immediately following introduction (early) with the period following attainment of widespread coverage (post). We chose a threshold of three-dose 75% coverage according to published evidence of reduced pneumonia rates at this level.[24] We conducted Lot Quality Assurance Surveys (LQAS) to determine vaccine coverage rates and the period at which such coverage was achieved.[25] LQAS is a sampling methodology that enables estimation of whether geographical areas, or lots, have vaccination coverage above a certain target threshold of desired coverage.[25] LQAS in rural Lilongwe estimated three-dose PCV13 coverage as 76% in children 6–16 months old by September 1st, 2013. We used a 6–16 month age range because of the catch-up campaign and our finding that third PCV13 doses were given at age 4–6 months on average and as late as 16 months.

### Data analysis

In descriptive univariable analyses we accounted for pneumonia seasonality by comparing the same six months in the early-PCV13 period (low coverage, January-June 2012) with the same six months post-PCV13 introduction (January-June 2014). We evaluated the association of monthly counts of pneumonia with three-dose PCV13 coverage more formally using quasi-Poisson time series regression methods to allow for temporally varying risk factors and autocorrelation [26, 27].

The main model fit was:

$$\begin{array}{l} Y_{t}∼\text{quasi-Poisson}(\mu_{t})\\ \;\text{log}(\mu_{t})={Β}_{0}+{Β}_{1}(3dose{}_{t})+{Β}_{2}({dres}_{t-1}) \end{array}$$

Where the subscript t is the time (month) to which the subscripted variable applies; Y_t_ is the count of pneumonia cases; quasi-Poisson denotes Poisson with overdispersion; *3dose*_t_ is the population three-dose PCV13 coverage, scaled from 0 to 1 so that 1 = 76% to give an estimated effect (B_1_) at 76% coverage, the level reached in the post-PCV13 period; and *dres*_t-1_ is the deviance residual for the previous month, t-1, in an identical model without the *dres* term in it, which was added to adjust for residual autocorrelation in the time-series [26, 28]. Gaussian models for log(Y_t_), which allow more standard approaches to allow for autocorrelation at the expense of not respecting as closely that the outcome comprised counts, were also explored in sensitivity analyses.

Age was not a potential confounder in these analyses, because age distribution in the total population changed minimally (<1%) over the period of study, and was therefore unrelated to population-level vaccine coverage. However, we did explore models with age as a potential effect modifier. We modelled age as a categorical variable in pre-specified groups of 0–5 months, 6–23 months and 24–59 months old based on age ranges which would be most likely to identify directly and indirectly attributable vaccine impact and because modelling age as a continuous variable resulted in model convergence problems. We report these models only as sensitivity analyses as they are underpowered and herd protection may cause auto-correlation across age groups and this would be difficult to model. Although our original intention was to allow for population changes of the period, because the changes did not significantly change the results, we preferred the simpler model without population adjustment. We include log(population) as an offset in sensitivity analyses.

We also explored controlling for seasonal patterns. The shortness of our data series made the usual approaches for this problematic [26], so we did this by including as an explanatory variable an estimate of annual seasonal pattern, from sine-cosine pairs of annual period and harmonics fitted to hospital data for Malawi from 2001 to 2012 (S3 Appendix) [20]. However, we found that coefficients for this variable fit to the 2012–14 data were either non-significant or, for danger sign cases and deaths, significantly negative, implying quite different seasonal patterns to that observed on average in 2001–12. In consequence, we retained this seasonal control only in sensitivity analyses.

All clinical pneumonia, fast breathing pneumonia, chest indrawing pneumonia, danger sign pneumonia, hypoxemic pneumonia (Table 1), and pneumonia hospital deaths were analysed in separate Poisson models. In addition, we estimated association of vaccine coverage with the proportion of pneumonia cases that had one or more clinical danger sign(s) with a logistic model for monthly values of this proportion, specified with the same explanatory variables as the above Poisson regression models.

We modelled the data from each level of the health system (hospitals, health centre and CHW) together in our main analyses, and separately in additional analyses. Missing data were few for clinical pneumonia and hypoxemia variables, and none for hospital vital status.

Stata 13.1 was used for all analyses, with the time-series regressions run using the *glm* command with *scale(x2)* option, on time-series set (*tsset*) data: the code for the main model for danger sign pneumonia being:

glm vsevere coverage76 L1.dres, family(poisson) scale(x2) eform.

We explored model fit with regression diagnostics and ran a total of 12 additional models as sensitivity analyses for each outcome, varying combinations of variables included in the model and assumptions relating to autocorrelation.

## Results

**Fig 2:**
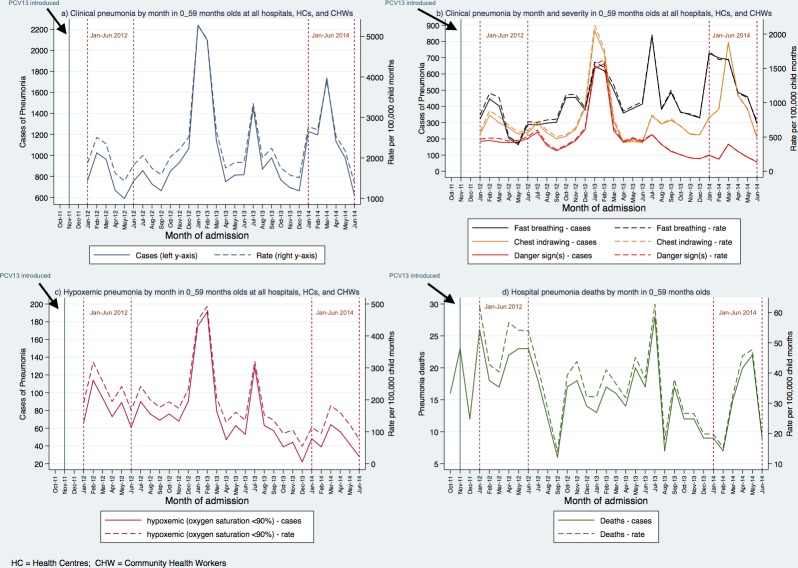
**Pneumonia cases and deaths in children under 5 years old by month: January 2012–June 2014:** a) Total clinical pneumonia cases at all hospitals, health centres and community health worker clinics b) Clinical pneumonia cases at all hospitals, health centres and community health worker clinics by category c) Hypoxemic pneumonia cases at all hospitals, health centres and community health worker clinics d) Hospital pneumonia deaths.

From January 1st, 2012-June 30th, 2014 a total of 30,630 cases of clinical pneumonia were recorded (Table 2). Three quarters of pneumonia patients (75.8%, n = 23,206) were <2 years. More than half of the patients we recorded were from the hospital level (53.8%, n = 16,475), while 22.1% (n = 6,764) were from health centres and 24.1% (n = 7,391) from CHWs. There were peaks of pneumonia cases in January, February and July 2013 and March 2014 (Fig 2).

**Table 2:** Clinical pneumonia, hypoxemic pneumonia, outcome, and PCV13 status by health system level in Lilongwe and Mchinji districts, Malawi

Health system level	Clinical pneumonia^1^				Hypoxemic pneumonia		Outcome		PCV13				
	Fast breathing only	Chest indrawing	Danger sign(s)	Missing	SpO_2_ Measured	SpO_2_<90%	Alive	Died	Recorded	0 doses	1 dose	2 doses	3 doses
All, n = 30630	13071 (42·7%)	10406 (34·0%)	5962 (19·5%)	1191 (3·9%)	27586 (89·3%)	2289 (8·3%)			22723 (74·2%)	6296 (27·7%)	2512 (11·1%)	2571 (11·3%)	11344 (49·9%)
Hospital, n = 16475	1049 (6·4%)	9254 (56·2%)	5397 (32·8%)	775 (4·7%)	14033 (85·2%)	1581 (11·3%)	15946 (96·8%)	529 (3·2%)	11297 (68·6%)	3304 (29·2%)	1469 (13·0%)	1324 (11·7%)	5200 (46·0%)
Health Centre, n = 6764	4786 (70·8%)	1082 (16·0%)	546 (8·1%)	350 (5·2%)	6312 (93·3%)	622 (9·9%)			5780 (85·5%)	1311 (22·7%)	640 (11·1%)	702 (12·1%)	3127 (54·1%)
CHW, n = 7391	7236 (97·9%)	70 (1·0%)	19 (0·3%)	66 (0·9%)	7241 (94·6%)	86 (1·2%)			5646 (76·4%)	1681 (29·8%)	403 (7·1%)	545 (9·7%)	3017 (53·4%)

SpO_2_ indicates peripheral oxygen saturation; CHW, community health worker. See S4 Appendix for breakdown by 0–5, 6–23 and 24–59 month age groups.

^1^See Table 1 for pneumonia definitions

Of children <5 years with PCV13 immunization records, 49.9% (n = 11,344/22,723) received all three doses. The highest proportion of cases fully immunized were 6–23 months old (71.7%, n = 8,567/11,948). Only 31.3% of cases 24–59 months (n = 1,336/4,266) and 22.1% aged 0–5 months (n = 1,441/6,509) received three doses (S4 Appendix).

Pneumonia was characterized by fast breathing only in 13,071 cases (42.7%), chest indrawing in 10,406 (34.0%) and clinical danger sign(s) in 5,962 (19.5%). Most cases with chest indrawing (88.9%, n = 9,254/10,406) or danger sign(s) (90.5%, n = 5,397/5,962) were hospitalized, while 92.0% (n = 12,022/13,071) with solely fast breathing were at health centres or with CHWs.

Eighty-nine percent of cases (n = 27,586/30,630) had a SpO_2_ measurement; 8.3% were hypoxemic. Cases 0–5 months had the highest hypoxemia prevalence (11.4%) compared to 6–23 month olds (8.4%) or 24–59 month olds (4.7%) (S4 Appendix). While the highest proportion of hypoxemic pneumonia patients were hospitalized (11.3%), 9.9% of health centre cases had hypoxemic pneumonia. Hypoxemia was infrequent in CHW-diagnosed children (1.2%). The mortality rate for cases hospitalized with pneumonia was 3.2%. Hospitalized cases with hypoxemic pneumonia had the highest mortality (8.3%) compared to pneumonia with danger sign(s) (5.4%), chest indrawing (2.1%), or only fast breathing (1.2%).

Table 3 shows the descriptive univariable analyses for pneumonia cases at all health system levels during January-June 2012 when three-dose PCV13 coverage was below 45% (early-PCV13) compared to January-June 2014 when three-dose coverage exceeded 76% (post-PCV13). Overall, there was an increase of 45% in all cases of clinical pneumonia. Fast breathing (+83%), and chest indrawing (+60%) pneumonia cases for children <5 years were higher during January-June, 2014, compared to January-June, 2012. These increases were mirrored by a 45% reduction in cases of danger sign pneumonia. Hypoxemic pneumonia cases also decreased 44%. Table 3 also disaggregates clinical and hypoxemic pneumonia rates into hospital, health centre, and CHW levels (see S5 Appendix for trends). Fast breathing pneumonia significantly increased at all levels and chest indrawing pneumonia increased by 322 cases/100,000 child months at hospitals (95% confidence interval (CI), 276, 368 cases/100,000 child months). Danger sign and hypoxemic pneumonia rates dropped at hospitals and health centres; and hypoxemic pneumonia rates also declined by 218 cases/100,000 child months in CHW-diagnosed patients (95% CI, 59, 377 cases/100,000 child months). Rates of pneumonia-associated mortality for hospitalized cases <5 years declined by 41% (-63%, -21%).

**Table 3:** Changes in case burden of clinical and hypoxemic pneumonia in children (0–59 months)

Outcome^1^	Health system level	January to June 2012 Total cases	January to June 2014 Total cases	Case difference	% change	p-value^1^
All clinical pneumonia^2^	All	4765	6886	2121	45%	<0·0001
Fast breathing pneumonia^2^	All	1828	3354	1526	83%	<0·0001
Chest indrawing pneumonia^2^	All	1608	2567	959	60%	<0·0001
Danger sign pneumonia^2^	All	1116	609	-507	-45%	<0·0001
Hypoxemic pneumonia^2^	All	497	277	-220	-44%	<0·0001
		Rate (cases/100,000 child months)	Rate (cases/100,000 child months)	Rate difference (95% CI)	% change (95% CI)	p-value
All clinical pneumonia	Hospital	1067	1263	196 (138, 255)	18% (13%, 24%)	<0·0001
	Health Centre	1615	2113	498 (360, 636)	31% (22%, 39%)	<0·0001
	CHW	13806	24857	11050 (9795, 12305)	80% (71%, 89%)	<0·0001
Fast breathing pneumonia	Hospital	53	120	67 (51, 83)	127% (97%, 157%)	<0·0001
	Health Centre	1064	1644	580 (462, 698)	54% (43%, 66%)	<0·0001
	CHW	13137	23341	10204 (8975, 11433)	78% (68%, 87%)	<0·0001
Chest indrawing pneumonia	Hospital	556	878	322 (276, 368)	58% (50%, 66%)	<0·0001
	Health Centre	324	267	-56 (-112, -1)	-17% (-35%, 0%)	0.0464
	CHW	126	196	71 (-58, 199)	56% (-46%, 159%)	0.2859
Danger sign pneumonia	Hospital	407	199	-208 (-238, -177)	-51% (-59%, -44%)	<0·0001
	Health Centre	158	92	-65 (-102, -29)	-42% (-65%, -18%)	0.0004
	CHW	28	78	51 (-23, 124)	181% (-82%, 445%)	0.1857
Hypoxemic pneumonia	Hospital	119	78	-40 (-58, -23)	-34% (-49%, -19%)	<0.0001
	Health Centre	252	75	-177 (-219, -135)	-70% (-87%, -54%)	<0·0001
	CHW	349	131	-218 (-377, -59)	-63% (-108%, -17%)	0.0063
Mortality	Hospital	52	31	-22 (-33, -11)	-41% (-63%, -21%)	0.0001

^1^See Table 1 for pneumonia definitions

^2^p-value estimated assuming a population equivalent to that of the catchment area for the hospitals (i.e. the whole of Mchinji and Lilongwe districts). Note that cases were recorded from health centres in about 28% of this population and with community health workers in about 3% of the total population so these p-values are approximate and we can’t accurately calculate confidence intervals (or rates; only cases are reported in this table)

Using multivariable time series regression we estimated the association of an increase of three-dose PCV13 coverage from zero to 76% with changes in risk of all clinical pneumonia, fast breathing, chest indrawing and danger sign pneumonia, hypoxemic pneumonia, mortality, and the proportion of clinical pneumonia cases that had danger signs, controlling for autocorrelation as explained in the methods. Table 4 shows the results of the models. There was a non-significant increase in the risk of all clinical pneumonia: incident rate ratio (IRR): 1.47, 95% CI: 0.87, 2.49, p = 0.154. While the risk of fast breathing pneumonia increased significantly (IRR: 2.35, 95% CI: 1.39, 3.97, p = 0.001), the risk of chest indrawing (IRR: 1.39, 95% CI: 0.73, 2.68, p = 0.320) and danger sign pneumonia (IRR: 0.64, 95% CI: 0.31, 1.30, p = 0.212) were not significantly associated with vaccine coverage. There were significant decreases in the risks of hypoxemic pneumonia, and the proportion of danger sign pneumonia associated with 76% three-dose PCV13 coverage. The risk of hypoxemic pneumonia was reduced by 47% (95% CI: 5%, 70%; p = 0.031), and the proportion of clinical pneumonia cases that had danger signs was lowered by 65% (95% CI: 46%, 77%; p<0.0001) at 76% vaccine coverage compared to 0%. Importantly, the risk of hospital pneumonia death was also significantly lower: IRR: 0.64, 95% CI: 0.42, 0.99, p = 0.047; Table 4).

**Table 4:** Time-series regression results with the association of 76% three-dose PCV13 coverage on pneumonia outcomes highlighted

	All clinical pneumonia ^a^					
Model and covariate ^a^	IRR (95%CI);					
	p-value					
*3dose* (B_1_)	1.469 (0.865, 2.493);					
	p = 0.154					
*L1*.*dres* (B_2_)	1.015 (1.004, 1.025);					
	p = 0.008					
	Fast breathing pneumonia ^a^	Chest indrawing pneumonia ^a^	Danger sign pneumonia ^a^	Hypoxemic pneumonia ^a^	Mortality ^a^	Proportion danger sign ^a^
Model and covariate ^a^	IRR (95%CI);	IRR (95%CI);	IRR (95%CI);	IRR (95%CI);	IRR (95%CI);	OR (95%CI);
	p-value	p-value	p-value	p-value	p-value	p-value
*3dose* (B_1_)	2.347 (1.389, 3.966);	1.393 (0.725, 2.678);	0.636 (0.313, 1.295);	0.532 (0.299, 0.945);	0.643 (0.416, 0.994);	0.351 (0.230, 0.536);
	p = 0.001	p = 0.320	p = 0.212	p = 0.031	p = 0.047	p<0.0001
*L1*.*dres* (B_2_)	1.009 (0.990, 1.028);	1.026 (1.008, 1.043);	1.042 (1.025, 1.059);	1.055 (1.018, 1.094);	1.029 (0.932, 1.135);	1.060 (1.041, 1.078);
	p = 0.379	p = 0.003	p<0.0001	p = 0.004	p = 0.572	p<0.0001

IRR = Incidence Rate Ratio; OR = Odds Ratio

^a^ *3dose* is the population three-dose PCV13 coverage, scaled from 0 to 1 so that 1 = 76% to give an estimated effect (B_1_) at 76% coverage, the level reached in the post-PCV13 period; *L1*.*dres* is the deviance residual for the previous month in an identical model without the *dres* term in it, which was added to adjust for residual autocorrelation in the time-series

Observations = 29; these are the 30 calendar months in time (January 2012 to June 2014) minus one due to the inclusion of the one-month lagged residual term *L1*.*dres*

The sensitivity analyses (S6 Appendix) indicate that the results in Table 4 are fairly robust to alternative specifications of the model. For all clinical pneumonia (Fig A6.1 in S6 Appendix) 10 of the 12 models estimated a non-significant increase, the two Gaussian models without autocorrelation estimated a significant increase; point estimates of the IRR ranged from 1.32 to 1.66 and 95%CI ranged from 0.76 to 3.02. For fast breathing pneumonia (Fig A6.2 in S6 Appendix) the 12 models estimated significant increases with 76% three-dose vaccine coverage, with point estimates of the IRR ranging from 2.11 to 3.09, and 95% CI spanning the range 1.31 to 4.84. For chest indrawing pneumonia the IRR point estimates of the 12 models ranged from 1.22 to 1.63, and all had confidence intervals spanning 1 (i.e. non-significant effects; Fig A6.3 in S6 Appendix). Ten of the 12 Poisson regression models for danger sign pneumonia estimated non-significant reductions in risk; the IRR point estimates for all models ranged from 0.41 to 0.76 and 95% CI span 0.13 to 2.11, with the Gaussian models with autocorrelation being least precise (Fig A6.4 in S6 Appendix). Of note the decline in risk of danger sign pneumonia was larger and statistically significant when the 2001–12 seasonal pattern was included despite its anomalous negative coefficient (IRR: 0.46, 95% CI: 0.22, 0.93, p = 0.031, Fig A6.4 in S6 Appendix). The difference is due to the seasonal peaks from the retrospective data estimated by the *season* covariate not coinciding with the January, February and July 2013 peaks in the danger sign pneumonia outcome in this study (top middle panel Fig A3.2 in S3 Appendix). For hypoxemic pneumonia, six of the 12 models estimated a significant reduction in risk with 76% three-dose PCV13 vaccine coverage, with IRR point estimates ranging from 0.40 to 0.63 and 95% CI spanning the range 0.21 to 1.26 (Fig A6.5 in S6 Appendix). Ten of the 12 mortality models estimated a significant reduction in mortality risk with IRR point estimates ranging from 0.55 to 0.70 and 95% CI spanning 0.34 to 1.08; the only models estimating a non-significant effect on mortality were models 6 and 7 –the Poisson ones with *season* and one and two month lagged residuals (Fig A6.6 in S6 Appendix). For the proportion of pneumonia that had danger signs (Fig A6.7 in S6 Appendix) all of the models estimated the IRR to be below 1, with point estimates ranging from 0.26 to 0.38 and 95% CI spanning 0.15 to 0.96.

At the hospital level the results were inline with the main results: only the hypoxemia decline was not significant (S7 Appendix). At the health centre level no significant changes were observed in any of the outcomes except for hypoxemic pneumonia, where there was a significant decline (IRR: 0.36, 95% CI: 0.16, 0.81, p = 0.014, S7 Appendix). At the CHW level, there were significant increases in all clinical pneumonia, fast breathing (98% of all clinical pneumonia observed at this level, Table 2) and danger sign pneumonia, though due to there being only 19 cases of the latter during the whole study period (Table 2) the danger sign models were very unstable (S7 Appendix).

The sensitivity analyses with age as an effect modifier in the main model are shown in S8 Appendix. There was a significant increase in all clinical pneumonia in 6–23 month olds. The increase in fast breathing pneumonia was in 6–23 and 24–59 month olds, but not 0–5 month olds; there was a significant increase in chest indrawing pneumonia in 6–23 month olds; a significant decrease in danger sign pneumonia in 24–59 month olds; significant decreases in hypoxemic pneumonia in 0–5 and 24–59 month olds; a significant decrease in mortality in 24–59 month olds; and significant decreases in the proportion of pneumonia cases that had danger signs in all three age groups (S8 Appendix).

## Discussion

Using large-scale active surveillance, this study describes the impact of routine PCV13 use on childhood pneumonia in a PCV-naïve, high pneumonia burden low-income country in sub-Saharan Africa. We found a non-significant increase in total pneumonia cases. We found this was comprised of a large and significant increase in the risk of fast breathing pneumonia (of approximately 135%) as well as a suggested decrease in pneumonia cases with danger signs (approximate 36% reduction, p<0.05 significant in some models tested), resulting in a 65% reduction in the proportion of danger sign pneumonia cases in children <5 years across the health system. We also found that PCV13 introduction in Malawi was associated with a 47% reduction in hypoxemic pneumonia and a 36% reduction in hospital pneumonia mortality. Using our model estimates and observed cases between September-2013 and June-2014 (when the vaccine was at sufficient coverage) we estimate that, if the associations were causal, PCV13 prevented approximately 386 medically attended hypoxemic cases, and 74 deaths in Lilongwe and Mchinji districts in Malawi (in 442,946 child months at risk, S2 Appendix). We have attempted to be conservative in our estimates as these numbers would be greater if we had assumed some effect of the vaccine at lower coverage before September 2013. Nevertheless, our results suggest that pneumonia cases are common and frequently severe, and that severe cases can be prevented with routine use of conjugate vaccines.

There is no gold standard diagnostic test for pneumonia etiology. As a result, pneumococcal vaccine trials in children have utilized surrogate measures like chest radiograph consolidation that favour specificity over sensitivity and are important for evaluating trial endpoints.[18] By contrast, we conducted a pneumonia surveillance study to assess vaccine impact in a real-world setting where the majority of patients receive healthcare but radiography and laboratory resources are few. In the absence of radiographic and laboratory measures, we utilized pulse oximetry and pre-defined, broadly accepted classifications for clinical pneumonia.

The Malawi pneumonia definitions were adapted from the WHO and are used throughout Africa; Malawian providers have utilized them for more than a decade.[22] The criteria are pragmatic for non-physicians and were designed to be highly sensitive to increase antibiotic treatment for children with possible bacterial pneumonia, who prior to these guidelines were often not treated with antibiotics. As a compromise to achieving high sensitivity these guidelines were knowingly permitted to have a low specificity due to clinical overlap with illnesses from other bacterial pathogens, viruses, or malaria.[29, 30] In spite of the low specificity of the WHO criteria, PCV has still been found to be efficacious against WHO-defined pneumonia in African trials.[16, 18] A PCV9 trial in South Africa reported 12% (95% CI, 4%-20%) efficacy against a combination of chest indrawing and danger sign pneumonia in HIV-infected and -uninfected children <5 years, but no efficacy against fast breathing pneumonia.[18] In the Gambia, PCV efficacy was 7% (1%-12%) against fast breathing and chest indrawing pneumonia (some of which also had danger signs) and 16% (3%-28%) against all-cause pneumonia mortality.[16] Our observed reductions in danger sign pneumonia, hypoxemic pneumonia, and hospital pneumonia mortality were noticeably larger than what was observed in these clinical trials. Diarrhea and dehydration are known risk factors for severe, life-threatening pneumonia in developing countries,[31] and rotavirus is a leading cause of diarrhea in Malawian children.[32] Since rotavirus vaccine was also introduced during this study’s time period (October 2012), we speculate that the overlapping introduction of rotavirus vaccine, and its reported 64% effectiveness against rotaviral diarrhea in Malawian children,[32] is likely to have contributed to the greater than expected reductions seen in this analysis. The incidence of malaria, which can also overlap with pneumonia [31], to the best of our knowledge, did not change during the study period. The coverage of the Hib vaccine also remained high and stable at around 93% during the study period [33]. These two potential confounding factors therefore are unlikely to have contributed to the observed reductions in pneumonia incidence and mortality.

Interestingly, after full introduction of PCV we also observed an increase in lower risk clinical pneumonia cases that lacked danger signs. This paradoxical increase in milder clinical pneumonia in children (or reduced vaccine effectiveness for mild clinical pneumonia) has been previously observed in other PCV studies from the United States,[34] Philippines,[35] and South Africa.[18] We do not think that this finding in our study was due to improved surveillance over time as we maintained the same standardized pneumonia case definitions and supportive supervision practices throughout the study, and conducted a conservatively long three month pilot period to ensure there was standard data collection. Rather, a secondary analysis of the South African trial, along with the growing body of evidence from south Asia that most fast breathing pneumonia cases are likely of viral etiology and may not require antibiotic treatment at all,[36, 37] offer potential insight to this finding. The South African trial showed that following PCV9 introduction there was a decrease in hospitalized viral respiratory illnesses, and the authors deduced from this that a large proportion of severe pneumococcal pneumonia develops secondarily from viral infections.[38] Taking this into account, our findings may reflect a PCV13-induced shift away from higher-risk viral/pneumococcal combined disease towards a pneumococcal-free, lower-risk viral process that still meets the Malawi and WHO fast breathing or chest indrawing pneumonia definitions (see Table 1). From a health system perspective our data further demonstrates that PCV introduction in similar African settings is unlikely to be associated with less medically attended clinical pneumonia episodes overall (indeed our data suggests it was associated with more total cases), but could result in fewer high-risk pneumonia medical encounters and hospital deaths. The shift toward less severe disease could also be accompanied by changing seasonal patterns as suggested by our comparison with retrospective pre-PCV13 hospital data (S3 Appendix).

The observed reductions in severe disease may also reflect both direct and indirect PCV effects. Although vaccine coverage estimates were low among cases 24–59 months (32.6%), we estimated considerable reductions in rates of danger sign pneumonia (77%) and hypoxemic pneumonia (83%) in this age group. Similarly, among 0–5 month olds (PCV13 coverage: 22.1%), hypoxemic pneumonia rates decreased 43% (S8 Appendix). Other studies have reported indirect effects of PCV on clinical pneumonia, but mainly in unvaccinated adults.[4, 6] Nasopharyngeal carriage studies, however, suggest that vaccination of children reduces transmission of vaccine-type pneumococci to their unvaccinated contacts.[5, 14] A recent study reported a reduction in vaccine-type pneumococcal carriage prevalence among unvaccinated children two years after vaccine introduction, an observation that the authors attributed to herd protection.[14] Although our study did not assess colonization, the observed reductions in disease occurred over a similar timeframe and in a similar patient population, suggesting that herd effects of PCV may have conferred indirect protection to unvaccinated cases. As previously mentioned, rotavirus vaccine introduction may have also impacted the pneumonia disease patterns we observed. Notably, the interaction between rotavirus and pneumococcal conjugate vaccines on diarrheal and respiratory disease incidence and health outcomes in Malawian children is not well understood and is an active area of on-going research.

While the efficacy of PCV against pneumonia has been previously reported,[16, 18] to our knowledge no other studies have incorporated measures of SpO_2_. In African children with respiratory complaints an abnormally low SpO_2_ level can be associated with radiographic pneumonia, and SpO_2_<90% with mortality.[39] Amongst <5 year olds, we found a 47% reduction in hypoxemic pneumonia rates after PCV13 introduction. These objective SpO_2_ findings are consistent with the declines in subjectively assigned danger sign pneumonia. Given there are no oxygen resources at health centres, the 9.9% health centre hypoxemia prevalence is alarming. In addition, PCV13’s impact on hypoxemic pneumonia as a probe for pneumococcal pneumonia needs confirmation alongside validated endpoints in controlled studies.

Conducting a study of this design, scale and scope in a weak healthcare system is challenging. We lacked reliable baseline data prior to PCV13 introduction, although the slow increase in PCV13 three-dose coverage provided more low coverage data than expected. Under-ascertainment due to provider misdiagnosis is possible. However, our monthly supervision visits suggest misdiagnosis was likely negligible and did not change over time. While this surveillance system was extensive it was still intrinsically susceptible to under-ascertainment when care was not sought and also selection bias since we were only able to include children who accessed care. These biases should not affect the study findings since health-seeking behaviour is unlikely to have changed significantly during this relatively short study. Since our models were based on vaccination status at population level, which is complete for all cases, missing individual level vaccination data does not affect our analytic approach. 11% of cases were missing SpO_2_ data and hence could not be included in our hypoxemia models, and it is possible that such children in hospitals and health centres are sicker than those with SpO_2_ data [40]. We conducted an additional sensitivity analysis assuming these children were 50% more likely to have hypoxemia and found the main hypoxemia result to only change slightly from 0.532 (0.299, 0.945, p = 0.031; Table 4) to 0.550 (0.314, 0.962, p = 0.036). Despite extensive data collection efforts, our data lacked reliable personal identifiers, including addresses, so we were unable to adjust our analysis for area-level clustering or clustering within individuals (repeat admissions of the same patient for the same or subsequent illness). Our mortality analysis was limited to the hospital level as the health centre and CHW levels were not set-up to reliably collect information on treatment outcomes. Due to the restricted number of monthly data points we had insufficient power to control for potential confounding factors, including HIV status, which also lacked data, and malnutrition. We attempted to control for seasonal variation using retrospective data from Malawian hospitals, though seasonal peaks were not the same as in our data (S3 Appendix), and we adjusted our estimates for one-month lagged autocorrelation. A longer time series would have enabled us to better explore the influence of seasonal variation. We were unable to identify a suitable control for secular trends as childhood diseases under routine surveillance in Malawi either overlapped with clinical pneumonia (malaria)[30] or had a confounding intervention during the study period (rotaviral vaccine introduction October 2012 for diarrheal disease). Lastly, we assumed that LQAS data represented PCV13 coverage in the entire study area and this could be incorrect. However, healthcare in this sub-region is generally comparable and the assumptions made for our study time periods were conservative so that confounding effects from coverage differences are unlikely.

Other controlled and observational studies that include more specific pneumococcal pneumonia markers and are able to account for high-risk groups like HIV-infection and severe malnutrition are necessary to fully evaluate PCV13’s impact on Malawian children. This study only assessed the first two and a half years of PCV13 use. As such, continued surveillance for serotype replacement and long-term vaccine effectiveness remains important. Nevertheless, our study utilized widespread active surveillance to evaluate PCV13’s impact on Malawian childhood pneumonia case burden and incidence in little-studied urban and rural hospitals, outpatient health centres, and remote communities. After PCV13 was introduced we found an impressive reduction in hospital pneumonia fatality and the most severe forms of childhood pneumonia at all health system levels, providing strong support for PCV13 use in high burden African countries like Malawi.

## Supporting Information

S1 Appendix Pneumonia case recording forms: Hospital; Health Centre; Community Health Worker clinic (PDF)

S2 Appendix Population denominators (DOCX)

S3 Appendix Seasonal variation adjustment (PDF)

S4 Appendix Clinical pneumonia, hypoxemic pneumonia, outcome, and PCV13 status by age and health system level (PDF)

S5 Appendix Trend graphs for each health system level (PDF)

S6 Appendix Sensitivity analyses for main results (PDF)

S7 Appendix Hospital, HC and CHW results (PDF)

S8 Appendix Age category as effect modifier sensitivity analysis (PDF)

S1 Checklist STROBE Checklist (DOC)
