# Supplementary material for: Impact of the 13-Valent Pneumococcal Conjugate Vaccine on Clinical and Hypoxemic Childhood Pneumonia over Three Years in Central Malawi: An Observational Study
Source: PLoS One. 2017 Jan 4;12(1):e0168209. doi: 10.1371/journal.pone.0168209 (PMC5215454; doi:10.1371/journal.pone.0168209)
Supplement: S1 Appendix — (PDF) [file pone.0168209.s001.pdf]

VILLAGE CLINIC PNEUMONIA RECORDING FORM PatientID_________

Section A: VILLAGE CLINIC DETAILS

District: _____________________Health Facility: __________________________ Village Clinic: ___________________

Section B: PATIENT IDENTIFICATION DETAILS

Child's Name: __________________________Date of birth __/___/___ Sex: _Boy/Girl_ Diagnosis Date: __/__/___

Caregiver's Name: _______________________________ Relationship: Mother/Father/Other_____________

Physical Address: ________________________________ Village: _____________________ TA: ___________________

Section C: VACCINE STATUS

Tick (✓) if Yes and Cross (✗) if No. For PCV indicate dates of vaccine if yes			
**Age**	**Vaccines**		
**Birth**	BCG ☐	OPV-0 ☐	**PCV VACCINE DATES**
**6 weeks***	DPT-Hib+ HepB 1 ☐	OPV-1 ☐	PCV-1 ☐ Date__/___/____
**10 weeks***	DPT-Hib+ HepB 2 ☐	OPV-2 ☐	PCV-2 ☐ Date__/___/____
**14 weeks***	DPT-Hib+ HepB 3 ☐	OPV-3 ☐	PCV-3 ☐ Date__/___/____
**9 months**	Measles ☐		

Section D: SIGNS AND SYMPTOMS

Oxygen Saturation: ____% Respiratory Rate: _____ Heart Rate: _____ MUAC: ______cm Temperature_____°c

☐ Cough ☐ Fast breathing ☐ Chest in drawing ☐ Convulsion ☐ Difficult breathing ☐ Not feeding well
☐ Palmar Pallor ☐ Very Sleepy or Unconscious ☐ Swelling of both feet ☐ Vomiting everything ☐ Others__________

Note: Check all that apply

Section E: TREATMENT/ PRE-REFERAL TREATMENT

☐ **Refer to health facility**

Cotrimoxazole adult tablet- 80/400

☐ Age 2 months-12 months- ½ tablet
☐ Age 12 months-5 years- 1tablet

☐ **Treat at home**

Cotrimoxazole adult tablet- 80/400

☐ Age 2 months-12 months- ½ Tablet (total 5 tablets)
☐ Age 12 months-5 years- 1tablet (total 10 tablets)

Section F: FOLLOW-UP FEEDBACK

☐ Baby Alive ☐ Baby died Date:___/___/___: at Health Facility/ Home/ Others (Specify*)*: ____

☐ Follow up Feedback done on:____/____/______

Comments:____
____

Compiled By (HSA Name)____ Date: ______/_______/_______

Verified By (SHSA Name): _______________________________________ Date:_____/________/_______

Health Centre: Today's Date (dd/mm/yyyy):

PNEUMONIA HEALTH CENTRE RECORDING FORM

Name:

Address: ______________________ Village: ______________ TA: ______________

Age (months): __________ Sex (M/F): ______________

Number of days of signs/symptoms: More than 21 days ☐ Less than 21 days ☐

Antibiotic treatment prior to coming to health centre Yes ☐ No ☐ Self Referral ☐ Referral from village clinic ☐

Weight kg ____________ MUAC cm ______________

Temperature ^{0}C ________________ Respiratory rate per minute ________________

Oxygen Saturation ________________ Heart Rate per minute ______________

Healthcare provider name:

(tick cadre) CO ☐ MA ☐ HSA ☐ Nurse ☐

Healthcare provider signature:

Date (dd/mm/yyyy):

Previous pneumonia in the last 12 months Yes☐ No ☐

Previous hospital admissions for pneumonia in last 12 months Yes☐ No ☐

BCG: No ☐ Yes ☐

PCV: No ☐ Yes ☐ # Doses: _______

Polio: : No ☐ Yes ☐ # Doses: _______

DTP-HepB-Hib: No ☐ Yes ☐ # Doses: _______

measles: No ☐ Yes ☐

Clinical features

CHILD 2 MONTHS TO 5 YEARS

Clinical features		
Chest in-drawing	Yes ☐	No ☐
Grunting	Yes ☐	No ☐
Nasal flaring	Yes ☐	No ☐
Head nodding	Yes ☐	No ☐
Central cyanosis	Yes ☐	No ☐
Sleepy/difficult to wake	Yes ☐	No ☐
Convulsions	Yes ☐	No ☐
Not able to breastfeed	Yes ☐	No ☐
Not able to drink	Yes ☐	No ☐
Stridor in calm child	Yes ☐	No ☐
Wheeze	Yes ☐	No ☐

Classification		Referral Decision	Treatment
Very severe pneumonia	☐	Prepare for referral ☐	Benzylpenicillin 50,000 IU/kg/dose ☐
Severe pneumonia	☐	Prepare for referral ☐	Benzylpenicillin 50,000 IU/kg/dose ☐
Pneumonia	☐	Treat at home ☐	Cotrimoxazole 2-12 months (5 tabs) ☐ ***or***<br>Cotrimoxazole 12 months to 5 years (10 tabs) ☐
PCP	☐	Prepare for referral ☐	Benzylpenicillin 50,000 IU/kg/dose ☐<br>***and***<br>Cotrimoxazole ½ tab ☐
Other (specify)	☐		

YOUNG INFANT < 2 MONTHS

Clinical features		
Chest in-drawing	Yes ☐	No ☐
Grunting	Yes ☐	No ☐
Nasal flaring	Yes ☐	No ☐
Head nodding	Yes ☐	No ☐
Central cyanosis	Yes ☐	No ☐
Sleepy/difficult to wake	Yes ☐	No ☐
Not feeding well	Yes ☐	No ☐
Wheeze	Yes ☐	No ☐
Stridor (calm child)	Yes ☐	No ☐
Apnoeic spells	Yes ☐	No ☐
Convulsions	Yes ☐	No ☐

Classification		Referral Decision	Treatment
Very severe pneumonia	☐	Prepare for referral ☐	Benzylpenicillin 50,000 IU/kg/dose ☐
Severe pneumonia	☐	Prepare for referral ☐	Benzylpenicillin 50,000 IU/kg/dose ☐
PCP	☐	Prepare for referral ☐	Benzylpenicillin 50,000 IU/kg/dose ☐<br>***and***<br>Cotrimoxazole ¼ tab ☐
Other (specify)	☐		

HIV status	Positive ☐	Negative ☐	Exposed ☐	Unknown ☐	Measles at this visit or in past 2 months Yes ☐ No ☐
Blood film (malaria)	Positive ☐	Negative ☐	Unknown ☐		Severe malnutrition Yes ☐ No ☐

District Hospital

District Registration No:

PNEUMONIA INPATIENT RECORDING FORM

Name:

Address: ______________________________

Age (months): ________ Sex (M/F): ________

Number of days of signs/symptoms: More than 21 days ☐ Less than 21 days ☐

Antibiotic treatment prior to coming to hospital: Yes ☐ No ☐ Self referral ☐ Referred by Health Centre ☐

Date of hospital admission: ________

Weight kg ________ MUAC cm ________

Temperature ^{0}C ________ Respiratory rate x 1 minute ________

Oxygen Saturation ________ Heart Rate per minute ________

Chest Xray Yes ☐ No ☐

If yes date taken and results:

Previous pneumonia in the last 12 months Yes☐ No ☐

Previous hospital admissions for pneumonia in last 12 months Yes☐ No ☐

BCG: No ☐ Yes ☐

PCV: No ☐ Yes ☐ # Doses: ________

Polio: : No ☐ Yes ☐ # Doses: ________

DTP-HepB-Hib: No ☐ Yes ☐ # Doses: ________

MMR: No ☐ Yes ☐

Clinical features / Classification

CHILD 2 MONTHS TO 5 YEARS

Clinical features			Classification	
Chest in-drawing	Yes ☐	No ☐	Very severe pneumonia	☐
Grunting	Yes ☐	No ☐	Severe pneumonia	☐
Nasal flaring	Yes ☐	No ☐	Pneumonia	☐
Head nodding	Yes ☐	No ☐	PCP	☐
Central cyanosis	Yes ☐	No ☐	Other (specify)	☐
Sleepy/difficult to wake	Yes ☐	No ☐		
Convulsions	Yes ☐	No ☐		
Not able to breastfeed	Yes ☐	No ☐		
Not able to drink	Yes ☐	No ☐		
Stridor in calm child	Yes ☐	No ☐		
Wheeze	Yes ☐	No ☐		

Treatment

Antibiotic	Dose	Day 1	Day 2	Day 3	Day 4	Day 5	Day 6	Day 7
Benzylpenicillin								
Amoxycillin								
Chloramphenicol								
Cotrimoxazole								
Other antibiotic (specify)								
Other treatment	Oxygen							

YOUNG INFANT < 2 MONTHS

Clinical features			Classification	
Chest in-drawing	Yes ☐	No ☐	Very severe pneumonia/disease	☐
Grunting	Yes ☐	No ☐	Severe pneumonia	☐
Nasal flaring	Yes ☐	No ☐	PCP	☐
Head nodding	Yes ☐	No ☐	Other (specify)	☐
Central cyanosis	Yes ☐	No ☐		
Sleepy/difficult to wake	Yes ☐	No ☐		
Not feeding well	Yes ☐	No ☐		
Wheeze	Yes ☐	No ☐		
Stridor (calm child)	Yes ☐	No ☐		
Apnoeic spells	Yes ☐	No ☐		
Convulsions	Yes ☐	No ☐		

Antibiotics	Dose	Day 1	Day 2	Day 3	Day 4	Day 5	Day 6	Day 7
Gentamicin								
Benzylpenicillin								
Amoxycillin								
Other antibiotic (specify)								
Other treatment	Oxygen							

HIV status Positive ☐ Negative ☐ Exposed ☐ Unknown ☐

Blood film (malaria) Positive ☐ Negative ☐ Unknown ☐

Measles at this visit or in past 2 months Yes ☐ No ☐

Severe malnutrition * (see below) Yes ☐ No ☐

- Severe malnutrition is visible severe wasting or oedema in both feet

please turn over

Hospitalisation

Duration of hospitalisation in either ____________ Hours ________________ Days

Admission diagnosis ______________________________ Discharge diagnosis ______________________________

Discharge and Follow-up

Course of antibiotics to be completed at home	Yes ☐	No ☐	Child returned for follow-up visit	Yes ☐	No ☐
Mother informed to return with child once antibiotics completed	Yes ☐	No ☐	Course of antibiotic completed**	Yes ☐	No ☐
			Child fully recovered**	Yes ☐	No ☐

Treatment Results

Treatment completed(1)	☐	Failure at 48 hrs (2)	☐	Failure at Day 5	☐
Left against advise(3)	☐	Transferred (4)	☐	Outcome unknown (5)	☐
Died within 24 hours of admission	☐	Died after 24 hours of admission	☐	**(See below for definitions)**	

Additional Remarks:

Rationale for Information/Recording System

When the decision is reached that the child has pneumonia and requires hospitalisation then the ***"Pneumonia Inpatient Recording Form"*** must be completed in addition to other forms that may be used, such as critical care pathways. The use of this form is a prerequisite of the Project providing the drugs for treatment of such cases. The form is initiated when the patient is started on treatment and is completed on discharge. The form is provided to assist the health worker in providing good quality care for the patient. All information is transferred to the ***Pneumonia Inpatient Register.***

* If NO then tick Outcome Unknown (5) in Treatment Results section

** If YES then child can be registered as Treatment Completed(1) in Treatment Results section

1. Course of antibiotics completed and child fully recovered
2. Treatment failure means: Worsening of fast breathing, or Worsening of chest in-drawing, or Development/persistence of abnormal sleepiness or difficulty in awakening, or development/persistence of inability to drink or poor breastfeeding.
3. Child removed from the hospital against medical advise before treatment is completed
4. Child is referred for treatment to another health facility and the result of treatment is unknown; where the result is known, that result should be recorded in place of the result "transferred
5. When mother does not return with child for follow-up visit once course of antibiotic(s) is finished
