# Supplementary material for: Impact of the 13-Valent Pneumococcal Conjugate Vaccine on Clinical and Hypoxemic Childhood Pneumonia over Three Years in Central Malawi: An Observational Study
Source: PLoS One. 2017 Jan 4;12(1):e0168209. doi: 10.1371/journal.pone.0168209 (PMC5215454; doi:10.1371/journal.pone.0168209)
Supplement: S2 Appendix — (DOCX) [file pone.0168209.s002.docx]

**Appendix 2 Population Denominators**

We estimated the study population as of January 2011, and how the population grew during the analysis period (January 2012 to June 2014), using publically available population data.

|  | Total population July 2008^1^ | Annual growth rate^2^ | Est. Total population Jan 2011 |
| --- | --- | --- | --- |
| Mchinji - Hosp & HC | 456416 | 0.035 | 496352 |
| Mchinji - VC |  | 0.035 | 38000 |
| Kabadula - HC | 212095 | 0.031 | 228532 |
| Kabadula - VC |  | 0.031 | 38000 |
| KCH - Hospital | 1906272 | 0.044 | 2115962 |

^1^ From Table 7 of Malawi main census 2008 report, Population Size and Composition statistical tables available at <http://www.nsomalawi.mw/index.php?option=com_content&view=article&id=107%3A2008-population-and-housing-census-results&catid=8&Itemid=6> (accessed 19^th^ October 2016)

^2^ From Table 2.2 of Malawi main census 2008 report, available at <http://www.nsomalawi.mw/index.php?option=com_content&view=article&id=107%3A2008-population-and-housing-census-results&catid=8&Itemid=6> (accessed 19^th^ October 2016)

The hospital and health centre catchment populations for July 2008 are taken from the 2008 census: Mchinji = Mchinji district; Kabudula = Traditional Authority area (TA) Kabudula and TA Khongoni; and KCH (Kamuzu Central Hospital) = Lilongwe Rural and Lilongwe City. The village clinic populations are based on a pre-defined population of approximately 2000 per village clinic. The annual growth rates are intercensal, from 1998 to 2008. We assumed the same annual growth rate between 2008 and 2014 based on declining fertility accompanied by declining mortality, particularly from HIV/AIDS. Using the age breakdown reported in Table 4 of the census Population Size and Composition statistical tables gave us January 2011 populations of under 5s in person months (the denominator for our analyses) as follows:

| **January 2011** | 0-5 months | 6-23 months | 24-59 months | Total 0-59 months |
| --- | --- | --- | --- | --- |
| Mchinji - Hosp & HC | 829 | 2338 | 4645 | 7811 |
| Mchinji - VC | 61 | 172 | 342 | 575 |
| Kabudula - HC | 378 | 1065 | 2117 | 3560 |
| Kabudula - VC | 61 | 172 | 342 | 575 |
| KCH - Hospital | 3316 | 9351 | 18580 | 31247 |
| ALL Hospital | 4145 | 11688 | 23225 | 39058 |
| ALL Health Centre | 1207 | 3403 | 6762 | 11372 |
| ALL Village Clinic | 122 | 344 | 683 | 1149 |

We then used the annual growth rates above, converted to monthly growth rates (*m*) using the formula *m*=(((1+*a*)/1)^1/12^)-1 where *a* is the annual growth rate, to project populations in subsequent months. We assumed the monthly growth rates were the same throughout the year. The populations at the start and end of our 30-month analysis period, in January 2012, and June 2014 were:

| **January 2012** | 0-5 months | 6-23 months | 24-59 months | Total 0-59 months |
| --- | --- | --- | --- | --- |
| Mchinji - Hosp & HC | 858 | 2419 | 4807 | 8085 |
| Mchinji - VC | 63 | 178 | 354 | 595 |
| Kabudula - HC | 390 | 1099 | 2183 | 3671 |
| Kabudula - VC | 63 | 177 | 352 | 592 |
| KCH - Hospital | 3462 | 9762 | 19398 | 32622 |
| ALL Hospital | 4320 | 12181 | 24205 | 40706 |
| ALL Health Centre | 1248 | 3518 | 6990 | 11756 |
| ALL Village Clinic | 126 | 355 | 706 | 1187 |
| **June 2014** | 0-5 months | 6-23 months | 24-59 months | Total 0-59 months |
| Mchinji - Hosp & HC | 932 | 2629 | 5224 | 8786 |
| Mchinji - VC | 69 | 193 | 384 | 646 |
| Kabudula - HC | 419 | 1183 | 2350 | 3952 |
| Kabudula - VC | 68 | 191 | 379 | 638 |
| KCH - Hospital | 3842 | 10833 | 21525 | 36199 |
| ALL Hospital | 4774 | 13462 | 26749 | 44985 |
| ALL Health Centre | 1352 | 3812 | 7574 | 12737 |
| ALL Village Clinic | 136 | 384 | 763 | 1284 |
