# Supplementary material for: Impact of the 13-Valent Pneumococcal Conjugate Vaccine on Clinical and Hypoxemic Childhood Pneumonia over Three Years in Central Malawi: An Observational Study
Source: PLoS One. 2017 Jan 4;12(1):e0168209. doi: 10.1371/journal.pone.0168209 (PMC5215454; doi:10.1371/journal.pone.0168209)

Appendix 3: Adjustment for Seasonality

To adjust for seasonality in the pneumonia outcomes we used retrospective hospital data from throughout Malawi from 2001 to 2012 [1]. This data is from differing, and increasing, numbers of the 41 tertiary hospitals in Malawi, therefore whilst it can be used to assess seasonal variation in pneumonia cases and deaths it should not be used to assess trends in the total numbers of cases and deaths over time.

To determine seasonal variation we fitted *sin* and *cos* functions (Fourier series) to the data for each pneumonia outcome[i] in the retrospective dataset. We used the `fourier` command in Stata 13.1 [2] on a variable transforming the monthly date of admission to the degrees of a circle representing one year to do this, as described in the Stata .do file contained as a supplementary file to an overview of this approach [3]. The best fitting number of *sin* and *cos* function pairs (harmonics) for each pneumonia outcome was determined by fitting 1-8 pairs of harmonics in different Poisson regressions of the outcome against the date of admission. We used Likelihood Ratio (LR) tests to compare the fit (-2 * log likelihood) of each of the 6 different unique regressions for each outcome[ii] in pairwise combinations, comparing the simplest (0 pairs of harmonics) to most complex (6 pairs of harmonics) models. As an example, for danger sign(s) pneumonia we started with 1 harmonic pair of *sin* and *cos* terms in the model and compared it to a model with 2 pairs of *sin* and *cos* terms. Finding the model with 2 pairs to not be statistically significantly better (p was >0.05 for the LR test) to the model with 1 pair, we then compared the model with 1 pair to a model with 3 pairs of harmonics. This model with 3 pairs was found to be a significantly better fit (p<0.05 for LR test); we then compared this model to models with 4, 5 and 6 pairs in turn, not finding any of these an any better fit. Therefore the model with 3 pairs of *sin* and *cos* terms was the best fitting of the 6 estimating the seasonal variation in danger sign(s) pneumonia and we used this to calculate the linear predictor.

To calculate the linear predictor of the best fitting Fourier series we collapsed the data, summing the number of cases or deaths (or averaging the proportion of pneumonia cases that have danger signs) by month, and including the *sin* and *cos* terms used in the best fitting model. We then ran a Poisson regression of the pneumonia outcome against the *sin* and *cos* terms and monthly date and calculated the linear predictor from this model. The values of this linear predictor for 2012 were used as a covariate in the multivariable time series regression models of PCV13 effect on the pneumonia outcomes (they are the variable $\ln[ff_t]$ described in the methods section of the paper). We used the linear predictor for 2012 only as it was the only year that overlapped between the retrospective study and our prospective study. We applied the 2012 values of the linear predictor to 2013 and 2014, i.e. January 2013 and January 2014 were

---

[i] Fast breathing, chest indrawing or danger sign(s) clinical pneumonia, death, and proportion of pneumonia cases that have danger sign(s). Please note the retrospective dataset did not include hypoxemic pneumonia; we therefore used the seasonal variation in danger sign(s) pneumonia as a proxy in models of this outcome.

[ii] for all of the outcomes harmonic pairs 7 and 8 were identical to harmonic pair 6 i.e. they did not add anything to the Fourier series

assumed to have the same value as January 2012. This assumption of the same seasonal variation in 2013 and 2014 is reasonable given the fit of the seasonal variation from 2001 to 2012 (Figure A3.1) in form. Differences in magnitude should be ignored due to the incomplete capture of total cases in each month, and are ignored by applying the 2012 numbers to 2013 and 2014.

To visualise the fitted Fourier series for each outcome we plot the exponent of the linear predictor (bold line) on a scatter plot of the monthly value of the outcome as Figure A3.1 below.

Figure A3.2 below shows that the fit from the 2001-2012 data, however, does not map well onto the seasonal variation observed in 2012-2014 after the PCV13 vaccine was rolled-out. This lack of fit – confirmed by non-significant, or negative coefficients of the log fitted Fourier terms as mentioned in the paper – is why these terms were not used in our main time-series regression models.

References

1. Lazzerini M, Seward N, Lufesi N, Banda R, Sinyeka S, et al. (2016) Mortality and its risk factors in Malawian children hospitalised with clinical pneumonia, 2001 to 2012. Lancet Global Health 4: e57-68.
2. Statacorp (2013) Stata Statistical Software: Release 13. College Station, TX: StataCorp LP.
3. Bhaskaran K, Gasparrini A, Hajat S, Smeeth L, Armstrong B (2013) Time series regression studies in environmental epidemiology. Int J Epidemiol 42: 1187-1195.

Figure A3.1: Sine/cosine functions (Fitted Fourier series) for Pneumonia outcomes from Retrospective data from Hospitals in Malawi, 2001-2012

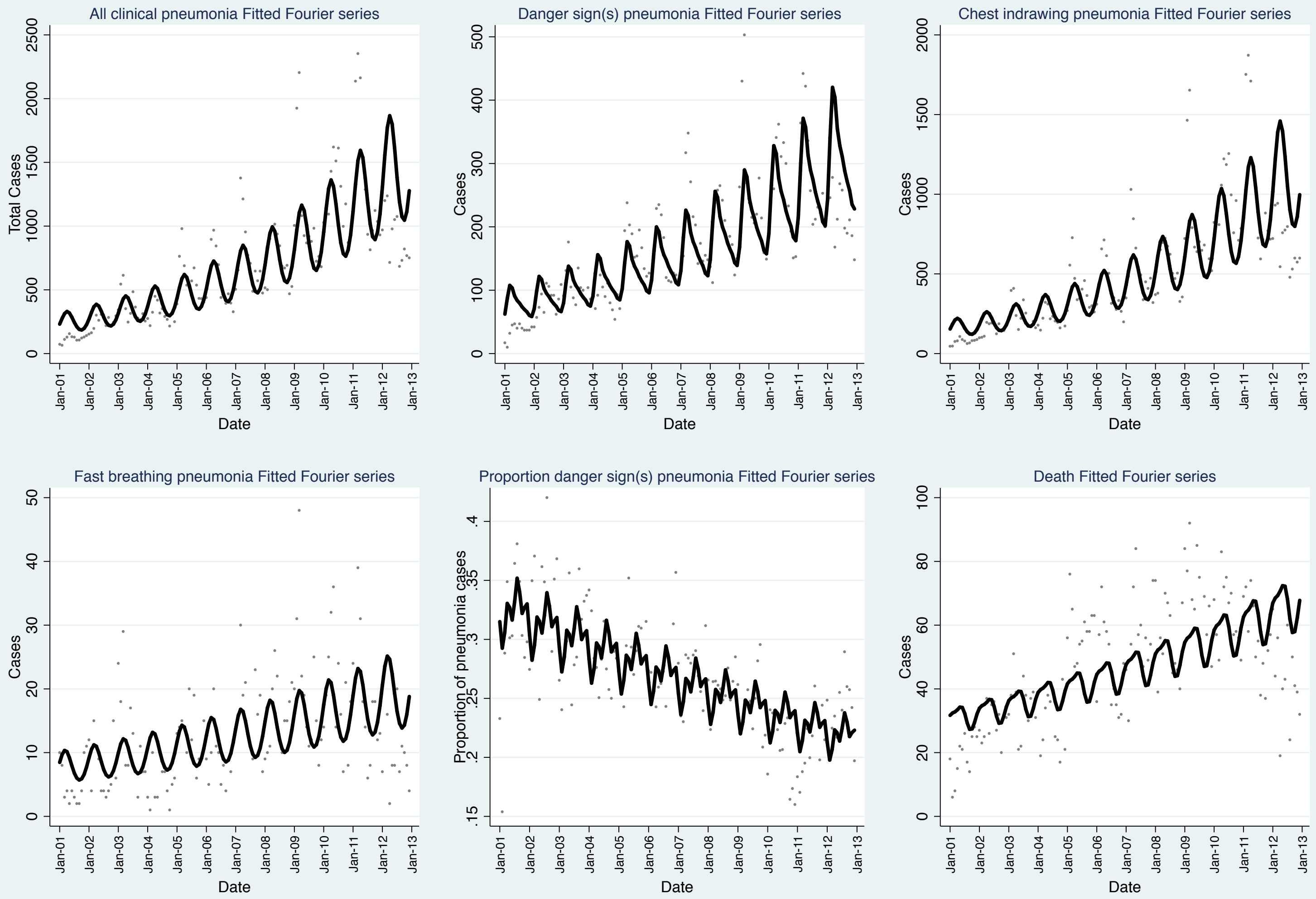

Figure A3.2: Seasonal variation from retrospective data (Log Fitted Fourier linear predictor) compared to Outcome trend

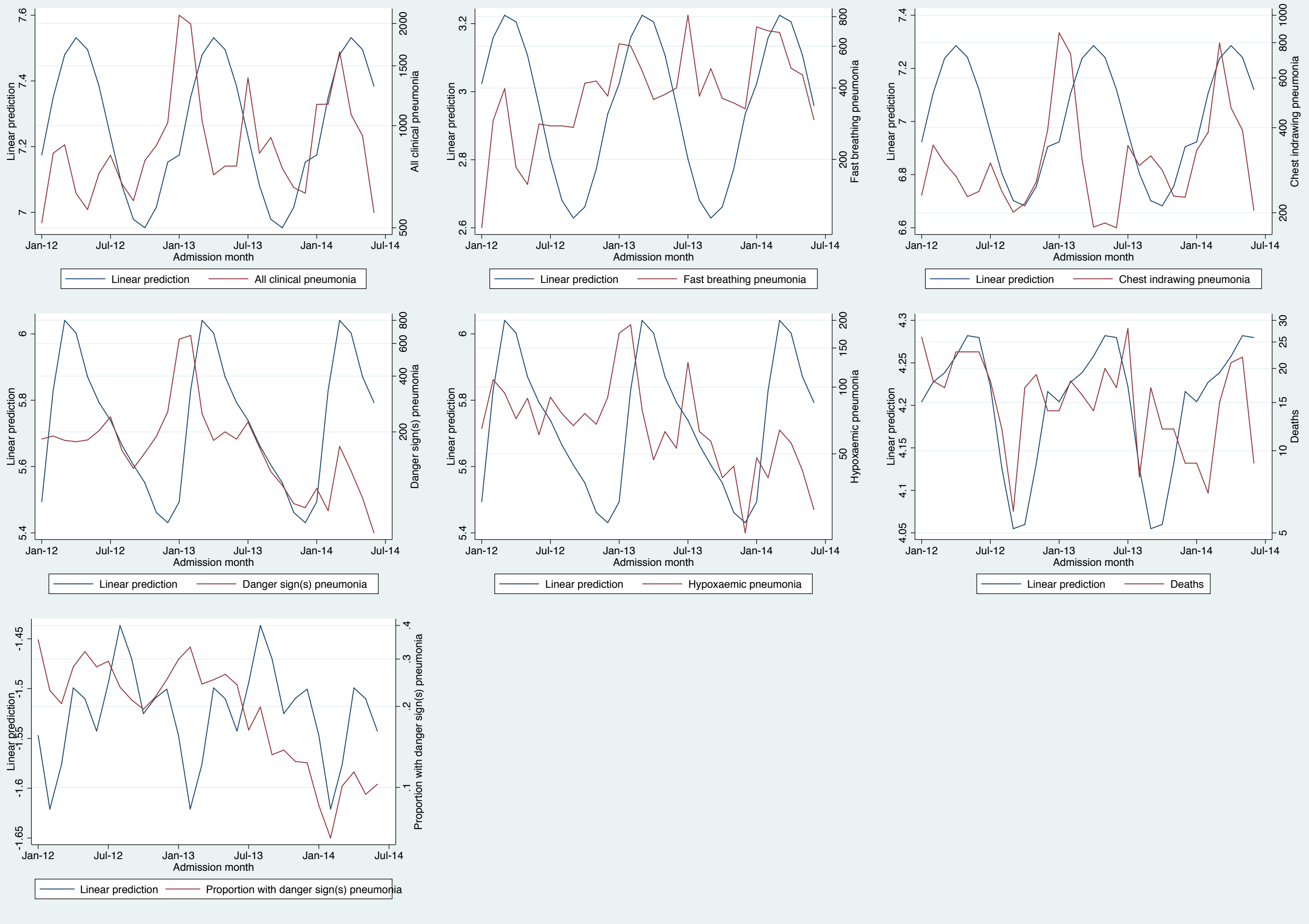

Supplement: S3 Appendix — (PDF) [file pone.0168209.s003.pdf]
