# Supplementary material for: Impact of the 13-Valent Pneumococcal Conjugate Vaccine on Clinical and Hypoxemic Childhood Pneumonia over Three Years in Central Malawi: An Observational Study
Source: PLoS One. 2017 Jan 4;12(1):e0168209. doi: 10.1371/journal.pone.0168209 (PMC5215454; doi:10.1371/journal.pone.0168209)
Supplement: S4 Appendix — (PDF) [file pone.0168209.s004.pdf]

Appendix 4 Clinical pneumonia, hypoxemic pneumonia, outcome, and PCV13 status by age and health system level

Table A4: Clinical pneumonia, hypoxemic pneumonia, outcome, and PCV13 status by age and health system level in Lilongwe and Mchinji districts, Malawi

Age range	Health system level	Clinical pneumonia[1]				Hypoxemic pneumonia		Outcome		PCV13				
		Fast breathing only	Chest indrawing	Danger sign(s)	Missing	SpO_2 Measured	SpO_2<90 %	Alive	Died	Recorded	0 doses	1 dose	2 doses	3 doses
0-5 months	All, n=7934	2015 (25·4%)	3276 (41·3%)	2182 (27·5%)	461 (5·8%)	7220 (91·2%)	826 (11·4%)			6509 (82·0%)	1857 (28·5%)	1800 (27·7%)	1411 (21·7%)	1441 (22·1%)
	Hospital, n=5104	251 (4·9%)	2832 (55·5%)	1996 (39·1%)	251 (4·9%)	4821 (90·5%)	606 (12·6%)	5135 (96·3%)	195 (3·7%)	4222 (82·7%)	1446 (34·2%)	1130 (26·8%)	839 (19·9%)	807 (19·1%)
	Health Centre, n=1534	823 (53·7%)	381 (24·8%)	186 (12·1%)	144 (9·4%)	1397 (91·1%)	193 (13·8%)			1376 (89·7%)	1061 (77·1%)	428 (31·1%)	316 (23·0%)	317 (23·0%)
	CHW, n=1070	941 (87·9%)	63 (5·9%)	0 (0·0%)	66 (6·2%)	1002 (94·9%)	27 (2·7%)			911 (85·1%)	815 (89·5%)	242 (26·6%)	256 (28·1%)	317 (34·8%)
6-23 months	All, n=15272	6614 (43·3%)	5197 (34·0%)	2948 (19·3%)	513 (3·4%)	13584 (88·1%)	1142 (8·4%)			11948 (78·2%)	1710 (14·3%)	608 (5·1%)	1063 (8·9%)	8567 (71·7%)
	Hospital, n=8222	543 (6·6%)	4660 (56·7%)	2651 (32·2%)	368 (4·5%)	6827 (83·0%)	777 (11·4%)	7973 (97·0%)	249 (3·0%)	5662 (68·9%)	956 (16·9%)	305 (5·4%)	446 (7·9%)	3955 (69·9%)
	Health Centre, n=3718	2756 (74·1%)	534 (14·4%)	283 (7·6%)	145 (3·9%)	3481 (93·6%)	324 (9·3%)			3259 (87·7%)	370 (11·4%)	181 (5·6%)	358 (11·0%)	2350 (72·1%)
	CHW, n=3332	3315 (99·5%)	3 (0·1%)	14 (0·4%)	0 (0.0%)	3276 (94·4%)	41 (1·3%)			3027 (90·8%)	384 (12·7%)	122 (4·0%)	259 (8·6%)	2262 (74·7%)
24-59 months	All, n=7424	4442 (59·8%)	1933 (26·0%)	832 (11·2%)	217 (2.9%)	6782 (89·7%)	321 (4·7%)			4266 (57·5%)	2729 (64·0%)	104 (2·4%)	97 (2·3%)	1336 (31·3%)
	Hospital, n=2923	255 (8·7%)	1762 (60·3%)	750 (25·7%)	156 (5.3%)	2385 (81·6%)	198 (8·3%)	2838 (97·1%)	85 (2·9%)	1413 (48·3%)	902 (63·8%)	34 (2·4%)	39 (2·8%)	438 (31·0%)
	Health Centre, n=1512	1207 (79·8%)	167 (11·1%)	77 (5·1%)	61 (4.0%)	1434 (94·8%)	105 (7·3%)			1145 (75·7%)	626 (54·7%)	31 (2·7%)	28 (2·4%)	460 (40·2%)
	CHW, n=2989	2980 (99·7%)	4 (0·1%)	5 (0·2%)	0 (0.0%)	2963 (94·8%)	18 (0·6%)			1708 (57·4%)	1201 (70·3%)	39 (2·3%)	30 (1·8%)	438 (25·6%)

SpO_2 indicates peripheral oxygen saturation; CHW, community health worker.

[1]See Panel in main paper for pneumonia definitions
