# Supplementary material for: Impact of the 13-Valent Pneumococcal Conjugate Vaccine on Clinical and Hypoxemic Childhood Pneumonia over Three Years in Central Malawi: An Observational Study
Source: PLoS One. 2017 Jan 4;12(1):e0168209. doi: 10.1371/journal.pone.0168209 (PMC5215454; doi:10.1371/journal.pone.0168209)

# Appendix 5

## Figure A5.1 Hospital Trends

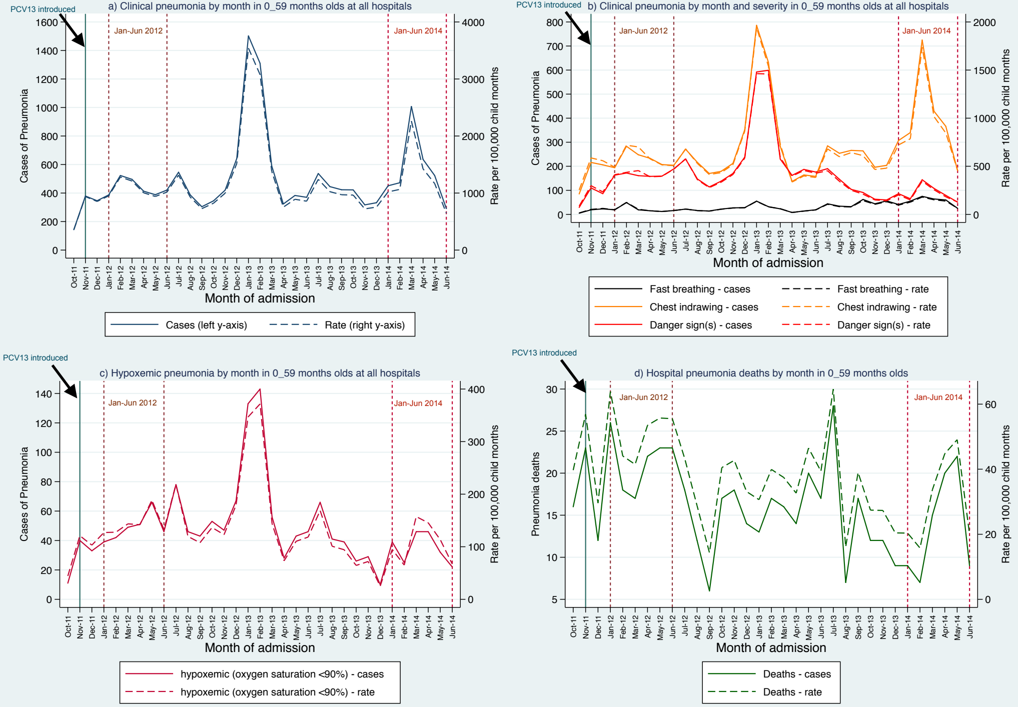

# Appendix 5

## Figure A5.2 Health Centre Trends

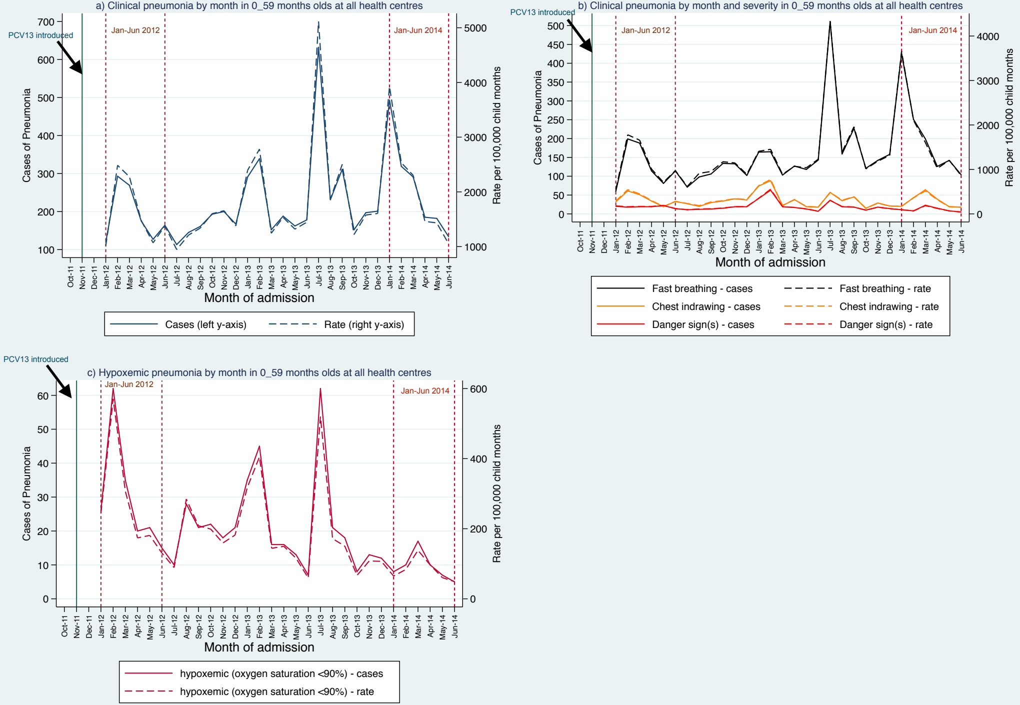

# Appendix 5

## Figure A5.3 Community Health Worker clinic Trends

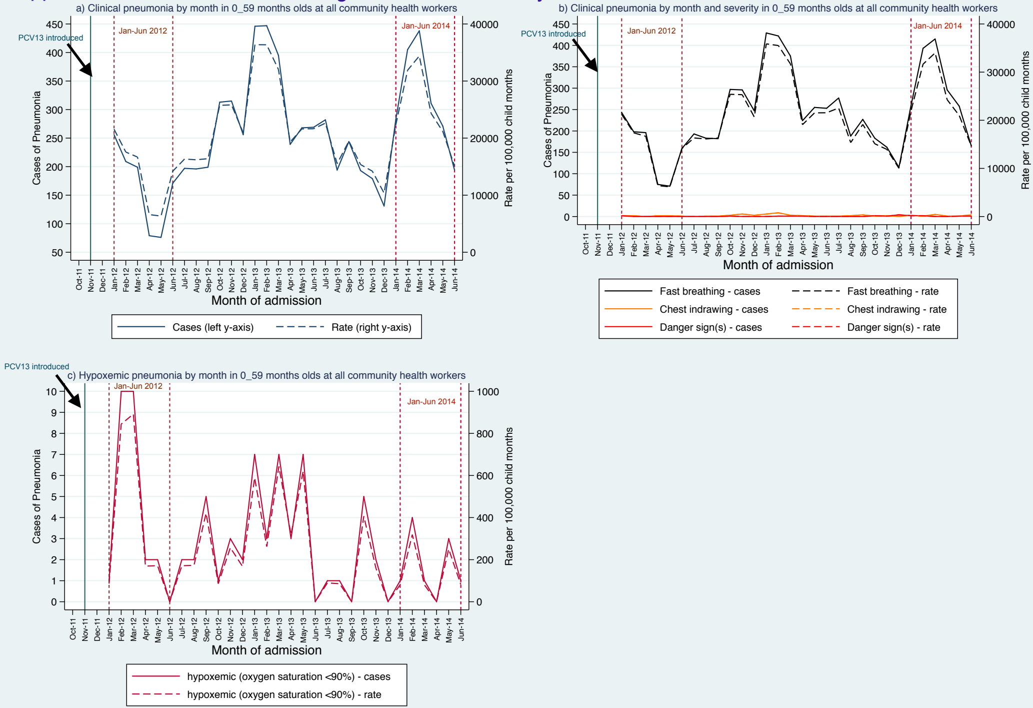

Supplement: S5 Appendix — (PDF) [file pone.0168209.s005.pdf]
