# Supplementary material for: Impact of the 13-Valent Pneumococcal Conjugate Vaccine on Clinical and Hypoxemic Childhood Pneumonia over Three Years in Central Malawi: An Observational Study
Source: PLoS One. 2017 Jan 4;12(1):e0168209. doi: 10.1371/journal.pone.0168209 (PMC5215454; doi:10.1371/journal.pone.0168209)

Appendix 6: Sensitivity Analysis Models for Main Results (Table 3 of paper)

Figures A6.1–A6.7 below show the results for the 12 sensitivity analyses for each of the seven main models reported in Table 3 of the paper. The 12 models for each of the seven outcomes are reported in the same order on each plot. From top to bottom they are:

1. “base no AC”: no seasonal variation variable or autocorrelation variables are added

2. “base AC1”: no seasonal variation variable added; autocorrelation with one month lag (AC1) added [this is the main model reported in Table 3 of the paper]

3. “base AC1 AC2”: no seasonal variation variable added; autocorrelation with one month lag (AC1) and autocorrelation with two months lag (AC2) added

4. “base AC1 pop offset”: no seasonal variation variable added; autocorrelation with one month lag (AC1) added; a offset for total population size (pop offset) added.

5. “season no AC”: seasonal variation variable added; no autocorrelation variables added

6. “season AC1”: seasonal variation variable added; autocorrelation with one month lag (AC1) added

7. “season AC1 AC2”: seasonal variation variable added; autocorrelation with one month lag (AC1) and autocorrelation with two months lag (AC2) added

8. “season AC1 pop offset”: seasonal variation variable added; autocorrelation with one month lag (AC1) added; a offset for total population size (pop offset) added

9. “Gaussian no AC”: Gaussian regression rather than Poisson regression; no seasonal variation variable added; no autocorrelation variables added

10. “Gaussian AC1”: Gaussian regression rather than Poisson regression; no seasonal variation variable added; autocorrelation with one month lag (AC1) added

11. “Gaussian season no AC”: Gaussian regression rather than Poisson regression; seasonal variation variable added; no autocorrelation variables added

12. “Gaussian season AC1”: Gaussian regression rather than Poisson regression; seasonal variation variable added; autocorrelation with one month lag (AC1) added

Please note that the models with each age group are separately presented as additional sensitivity analyses in Appendix 8

Figure A6.1 Sensitivity Analysis for All clinical pneumonia models

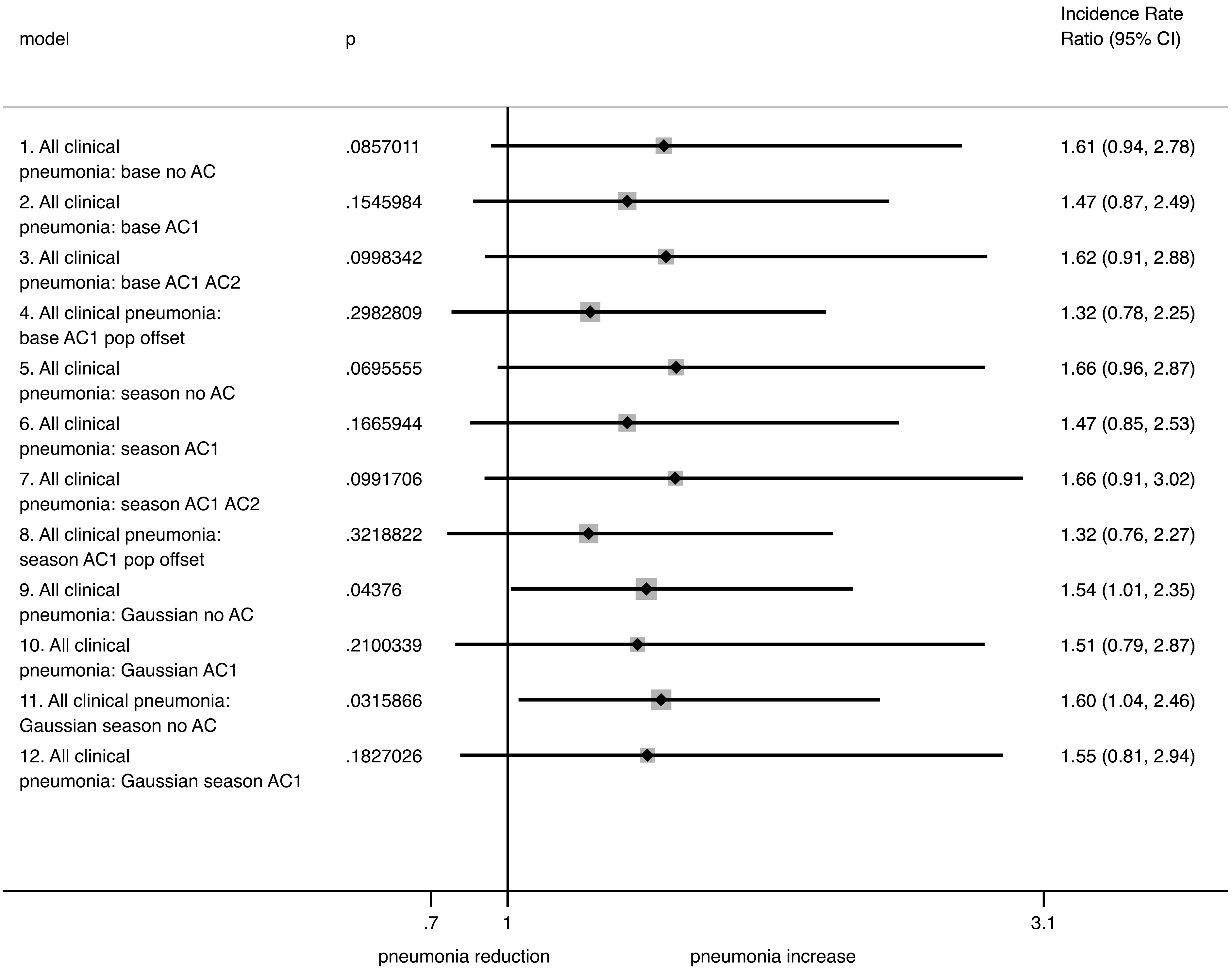

Figure A6.2 Sensitivity Analysis for Fast breathing pneumonia models

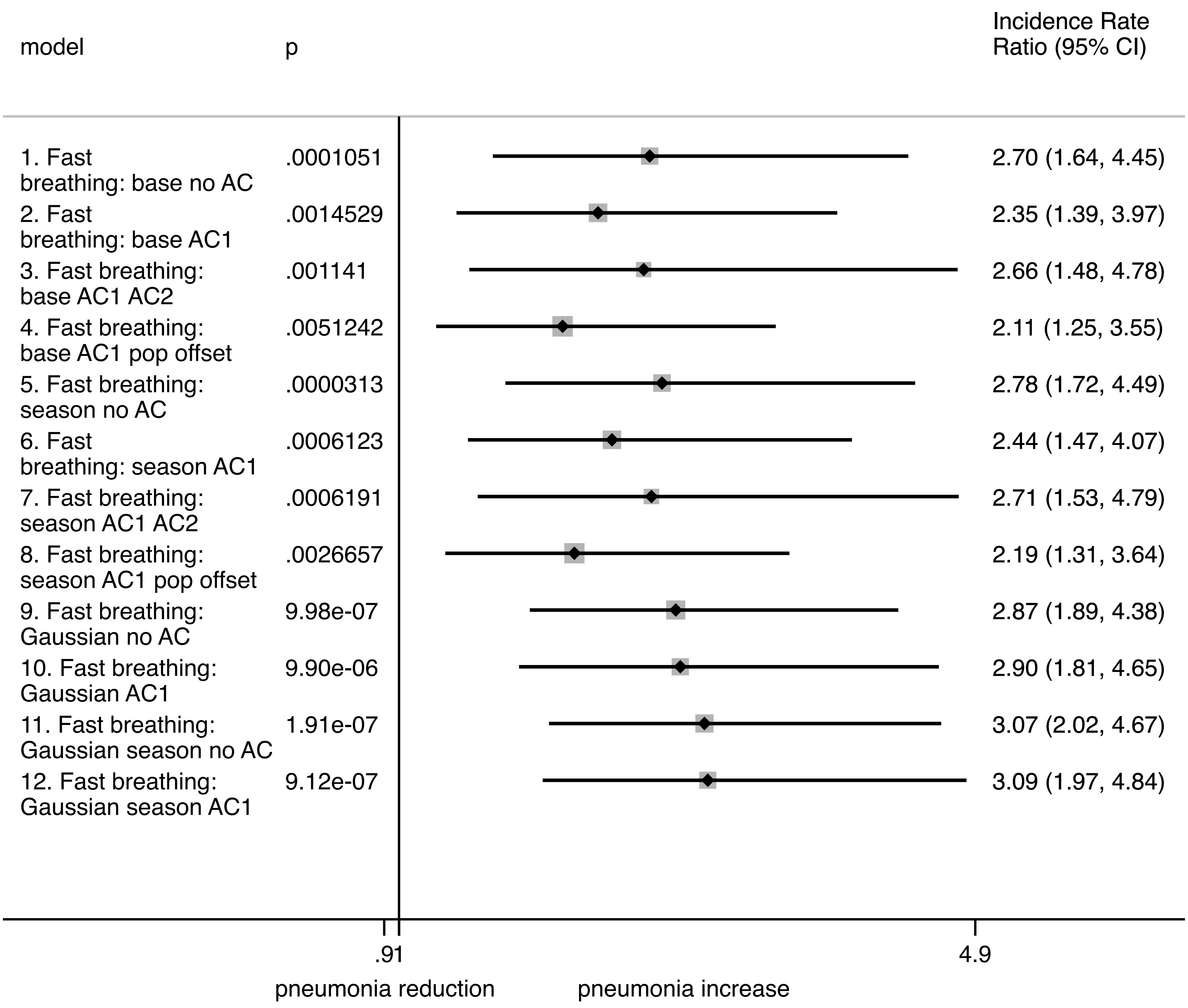

Figure A6.3 Sensitivity Analysis for Chest indrawing pneumonia models

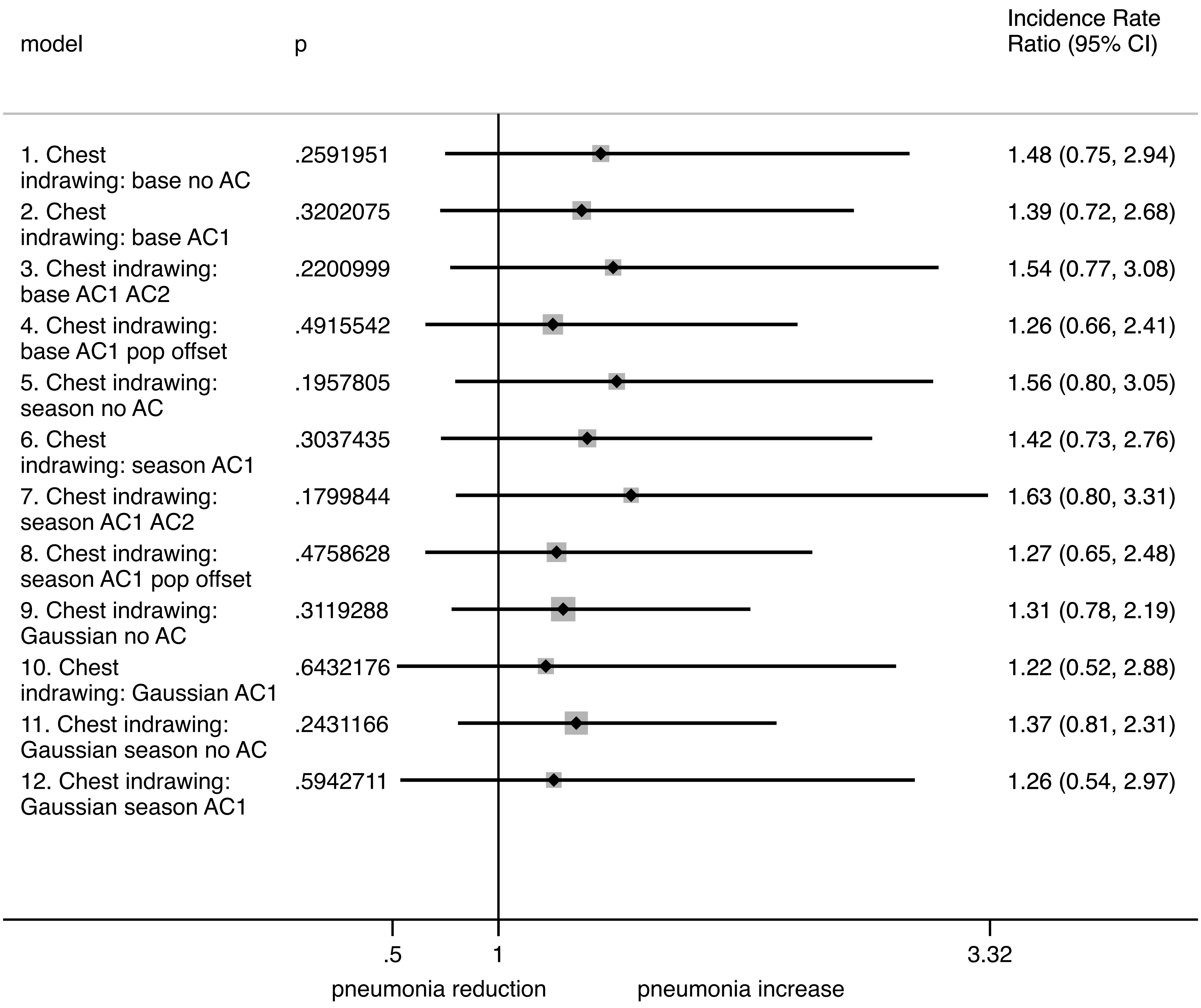

Figure A6.4 Sensitivity Analysis for Danger sign(s) pneumonia models

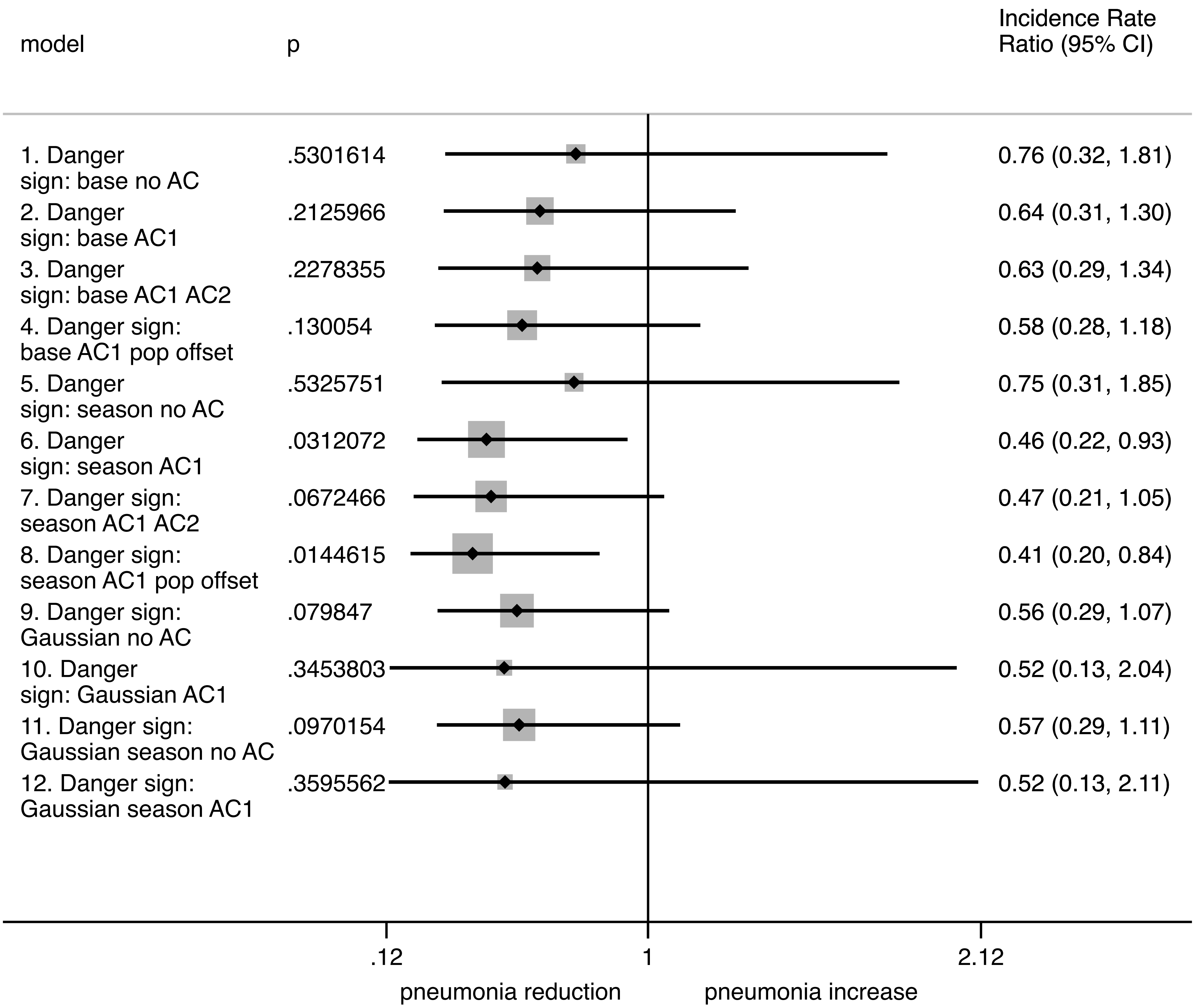

Figure A6.5 Sensitivity Analysis for Hypoxemic pneumonia models

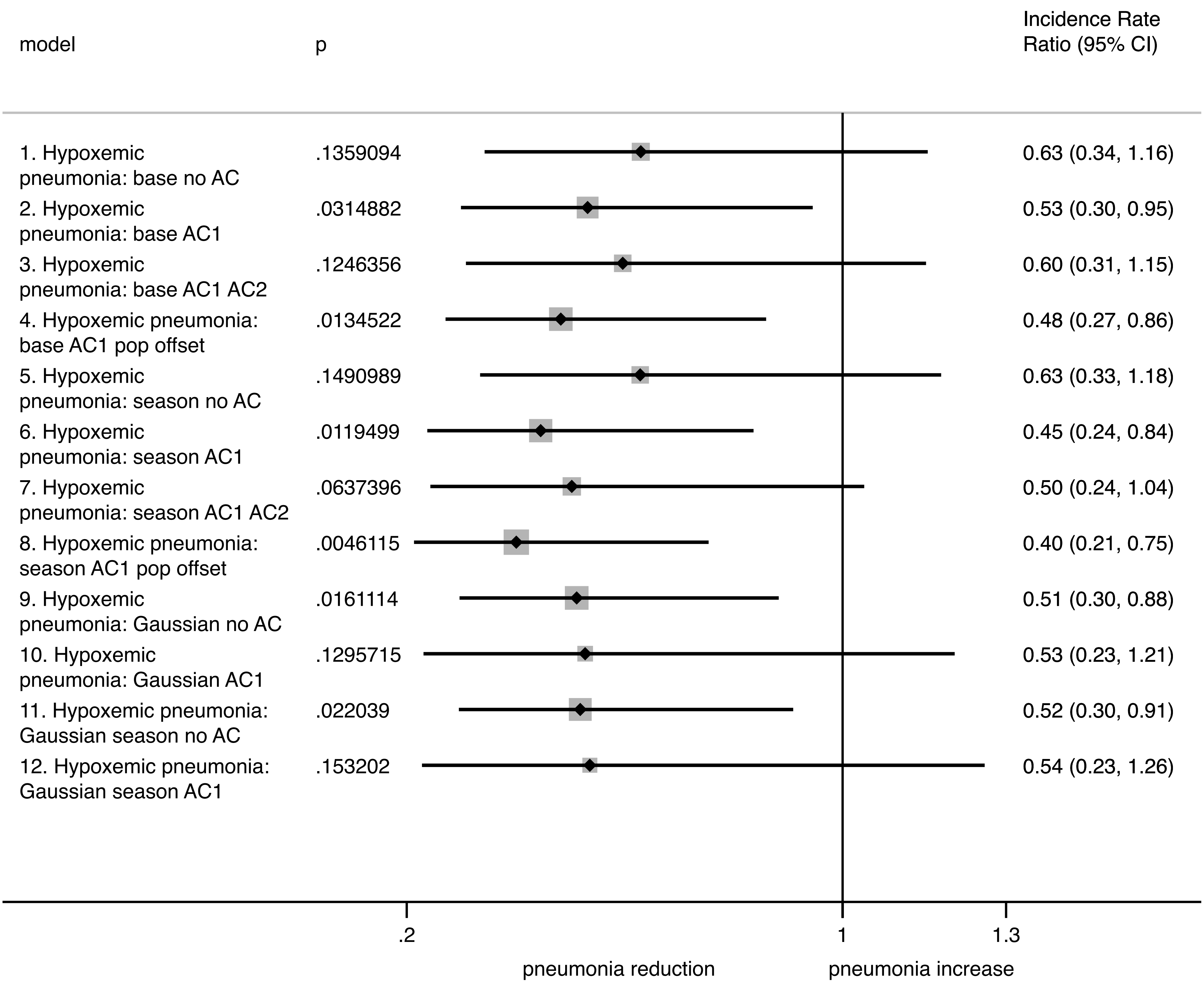

Figure A6.6 Sensitivity Analysis for Died pneumonia models

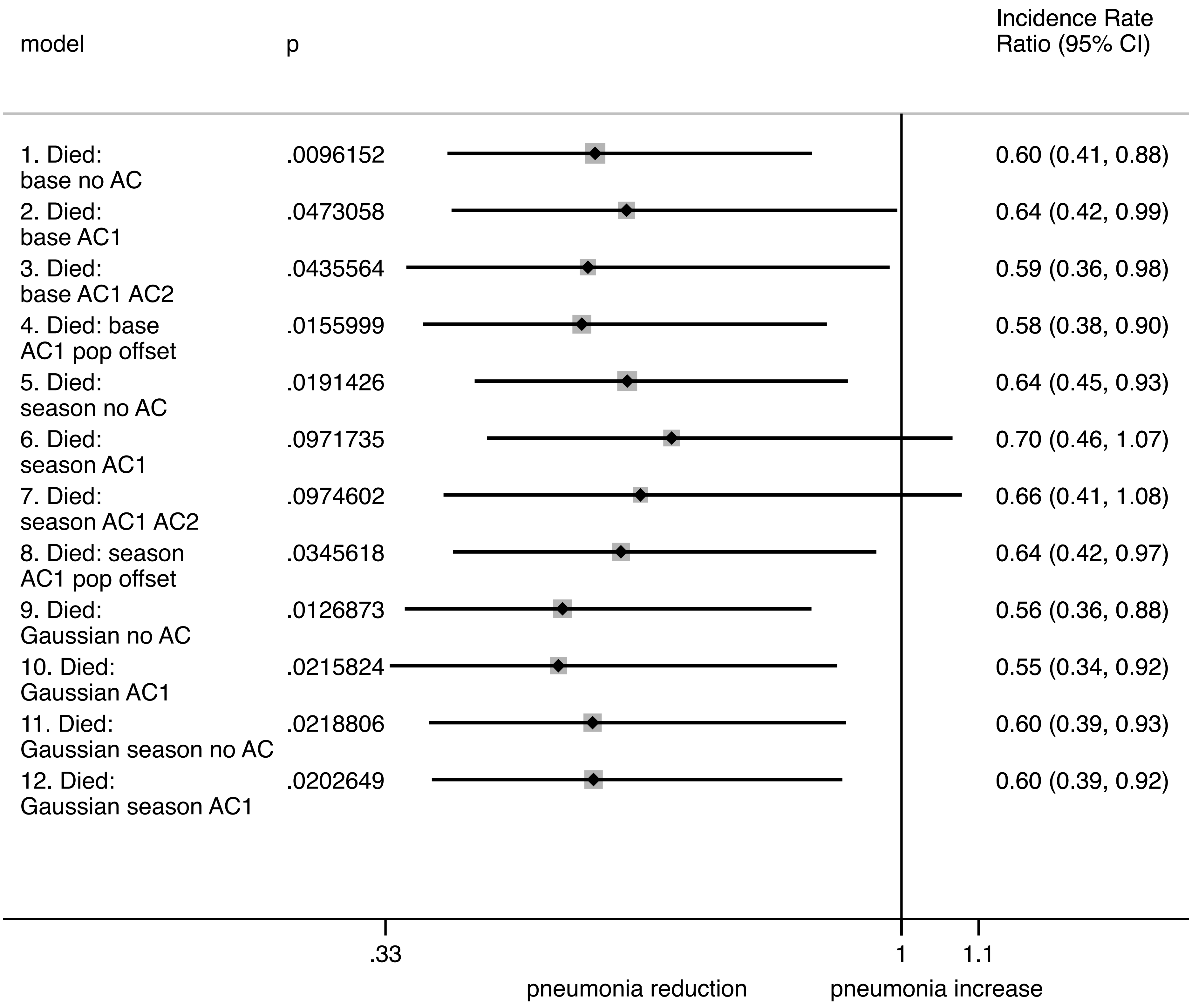

Figure A6.7 Sensitivity Analysis for proportion danger sign pneumonia models

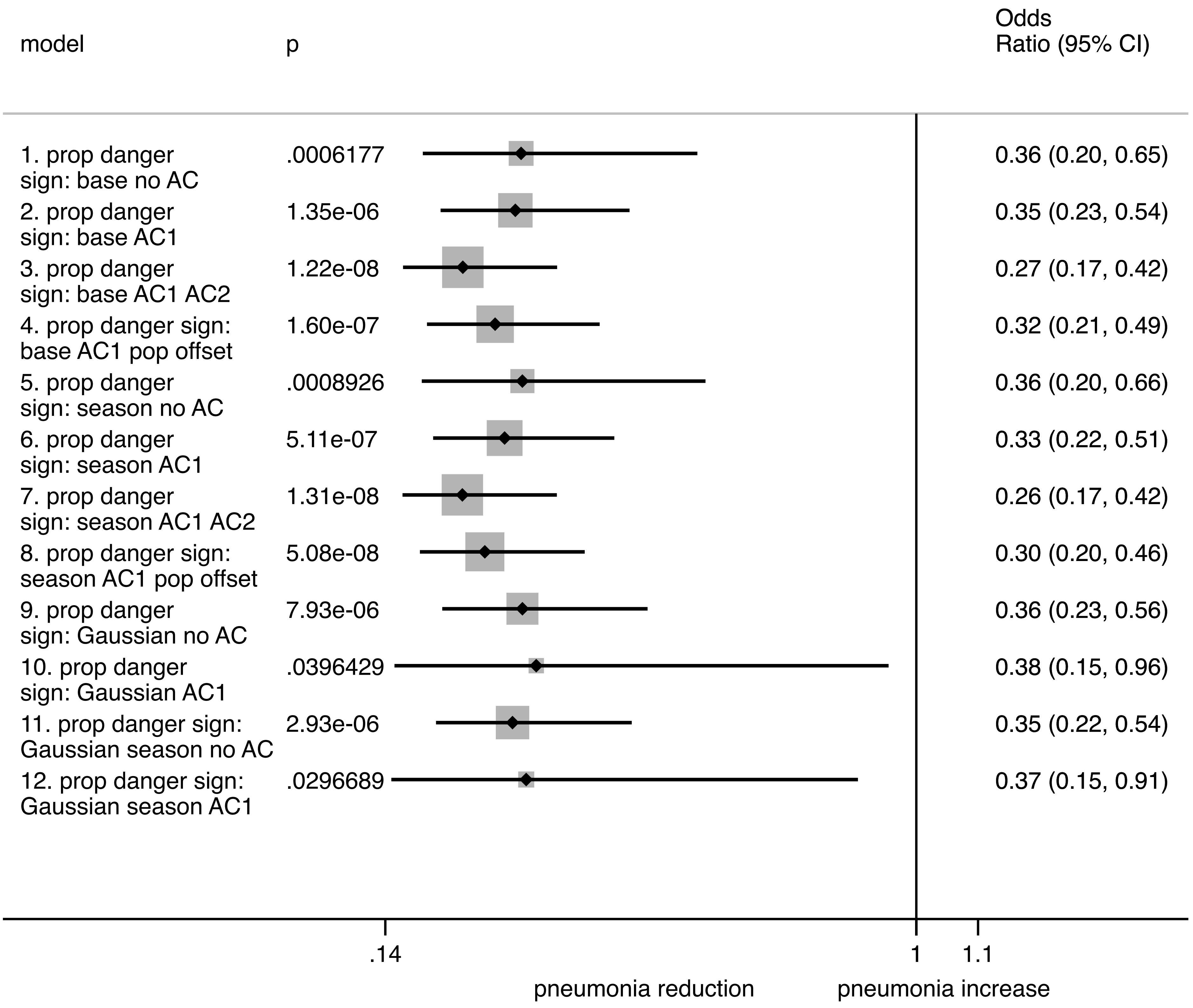

Supplement: S6 Appendix — (PDF) [file pone.0168209.s006.pdf]
