# Supplementary material for: Impact of the 13-Valent Pneumococcal Conjugate Vaccine on Clinical and Hypoxemic Childhood Pneumonia over Three Years in Central Malawi: An Observational Study
Source: PLoS One. 2017 Jan 4;12(1):e0168209. doi: 10.1371/journal.pone.0168209 (PMC5215454; doi:10.1371/journal.pone.0168209)

Appendix 7: Time-Series Regression Results for each Health System Level

Table A7.1. Hospitals: Time-series regression results with the association of 76% three-dose PCV13 coverage on pneumonia outcomes highlighted

Model and covariate [a]	All clinical pneumonia [a] IRR (95%CI); p-value
3dose (B_1)	1.154 (0.608, 2.192); p=0.661
L1.dres (B_2)	1.022 (1.009, 1.035); p=0.001
constant (B_0)	441.9 (267.8, 729.1); p<0.0001

Model and covariate [a]	Fast breathing pneumonia [a] IRR (95%CI); p-value	Chest indrawing pneumonia [a] IRR (95%CI); p-value	Danger sign pneumonia [a] IRR (95%CI); p-value	Hypoxemic pneumonia [a] IRR (95%CI); p-value	Mortality [a] IRR (95%CI); p-value	Proportion danger sign [a] OR (95%CI); p-value
3dose (B_1)	2.833 (1.387, 5.785); p=0.004	1.504 (0.759, 2.978); p=0.242	0.611 (0.294, 1.269); p=0.186	0.641 (0.347, 1.186); p=0.156	0.640 (0.412, 0.993); p=0.046	0.360 (0.256, 0.505); p<0.0001
L1.dres (B_2)	1.084 (1.015, 1.157); p=0.016	1.027 (1.009, 1.046); p=0.004	1.045 (1.027, 1.062); p<0.0001	1.074 (1.034, 1.117); p<0.0001	1.020 (0.924, 1.127); p=0.692	1.090 (1.072, 1.109); p<0.0001
constant (B_0)	14.71 (8.16, 26.53); p<0.0001	214.7 (124.7, 369.6); p<0.0001	231.8 (135.1, 397.7); p<0.0001	67.39 (42.64, 106.51); p<0.0001	21.42 (15.41, 29.77); p<0.0001	1.061 (0.822, 1.369); p=0.651

IRR = Incidence Rate Ratio   OR = Odds Ratio

[a] Observations = 29; these are the 30 calendar months in time (January 2012 to June 2014) minus one due to the inclusion of the one-month lagged residual term *L1.dres*

Table A7.2. Health Centres: Time-series regression results with the association of 76% three-dose PCV13 coverage on pneumonia outcomes highlighted

Model and covariate [a]	All clinical pneumonia [a] IRR (95%CI); p-value
3dose (B_1)	1.366 (0.667, 2.799); p=0.394
L1.dres (B_2)	1.005 (0.977, 1.034); p=0.727
constant (B_0)	172.7 (98.0, 304.3); p<0.0001

Model and covariate [a]	Fast breathing pneumonia [a] IRR (95%CI); p-value	Chest indrawing pneumonia [a] IRR (95%CI); p-value	Danger sign pneumonia [a] IRR (95%CI); p-value	Hypoxemic pneumonia [a] IRR (95%CI); p-value	Proportion danger sign [a] OR (95%CI); p-value
3dose (B_1)	1.664 (0.706, 3.921); p=0.244	0.781 (0.409, 1.491); p=0.453	0.840 (0.376, 1.874); p=0.670	0.360 (0.160, 0.811); p=0.014	0.671 (0.363, 1.242); p=0.204
L1.dres (B_2)	1.009 (0.977, 1.042); p=0.598	1.044 (0.982, 1.109); p=0.172	1.085 (1.002, 1.175); p=0.045	1.020 (0.928, 1.121); p=0.681	1.133 (1.052, 1.220); p=0.001
constant (B_0)	111.3 (56.1, 220.9); p<0.0001	43.65 (26.67, 71.44); p<0.0001	20.33 (11.02, 37.52); p<0.0001	41.50 (23.29, 73.94); p<0.0001	0.121 (0.075, 0.195); p<0.0001

IRR = Incidence Rate Ratio OR = Odds Ratio

[a] Observations = 29; these are the 30 calendar months in time (January 2012 to June 2014) minus one due to the inclusion of the one-month lagged residual term *L1.dres*

Table A7.3. Community Health Worker clinics: Time-series regression results with the association of 76% three-dose PCV13 coverage on pneumonia outcomes highlighted

Model and covariate [a]	All clinical pneumonia [a] IRR (95%CI); p-value
3dose (B_1)	2.991 (1.676, 5.337); p<0.0001
L1.dres (B_2)	1.034 (1.011, 1.057); p=0.004
constant (B_0)	97.75 (60.77, 157.2); p<0.0001

Model and covariate [a]	Fast breathing pneumonia [a] IRR (95%CI); p-value	Chest indrawing pneumonia [a] IRR (95%CI); p-value	Danger sign pneumonia [a] IRR (95%CI); p-value	Hypoxemic pneumonia [a] IRR (95%CI); p-value	Proportion danger sign [a] OR (95%CI); p-value
3dose (B_1)	2.969 (1.656, 5.324); p<0.0001	2.849 (0.747, 10.86); p=0.125	931.6 (6.844, 126788.2); p=0.006	0.437 (0.138, 1.381); p=0.158	769.7 (0.866, 683687.2); p=0.055
L1.dres (B_2)	1.033 (1.010, 1.057); p=0.004	1.164 (0.929, 1.459); p=0.187	1.332 (0.915, 1.938); p=0.134	0.995 (0.801, 1.238); p=0.967	1.309 (0.893, 1.920); p=0.167
constant (B_0)	97.09 (60.13, 156.8); p<0.0001	1.043 (0.350, 3.112); p=0.940	0.0018 (0.00002, 0.157); p=0.006	4.657 (2.012, 10.782); p<0.0001	8.0e-6 (1.7e-8, 0.004); p<0.0001

IRR = Incidence Rate Ratio OR = Odds Ratio

[a] Observations = 29; these are the 30 calendar months in time (January 2012 to June 2014) minus one due to the inclusion of the one-month lagged residual term *L1.dres*

Figure A7.1 Sensitivity Analysis for All clinical pneumonia models - Hospitals

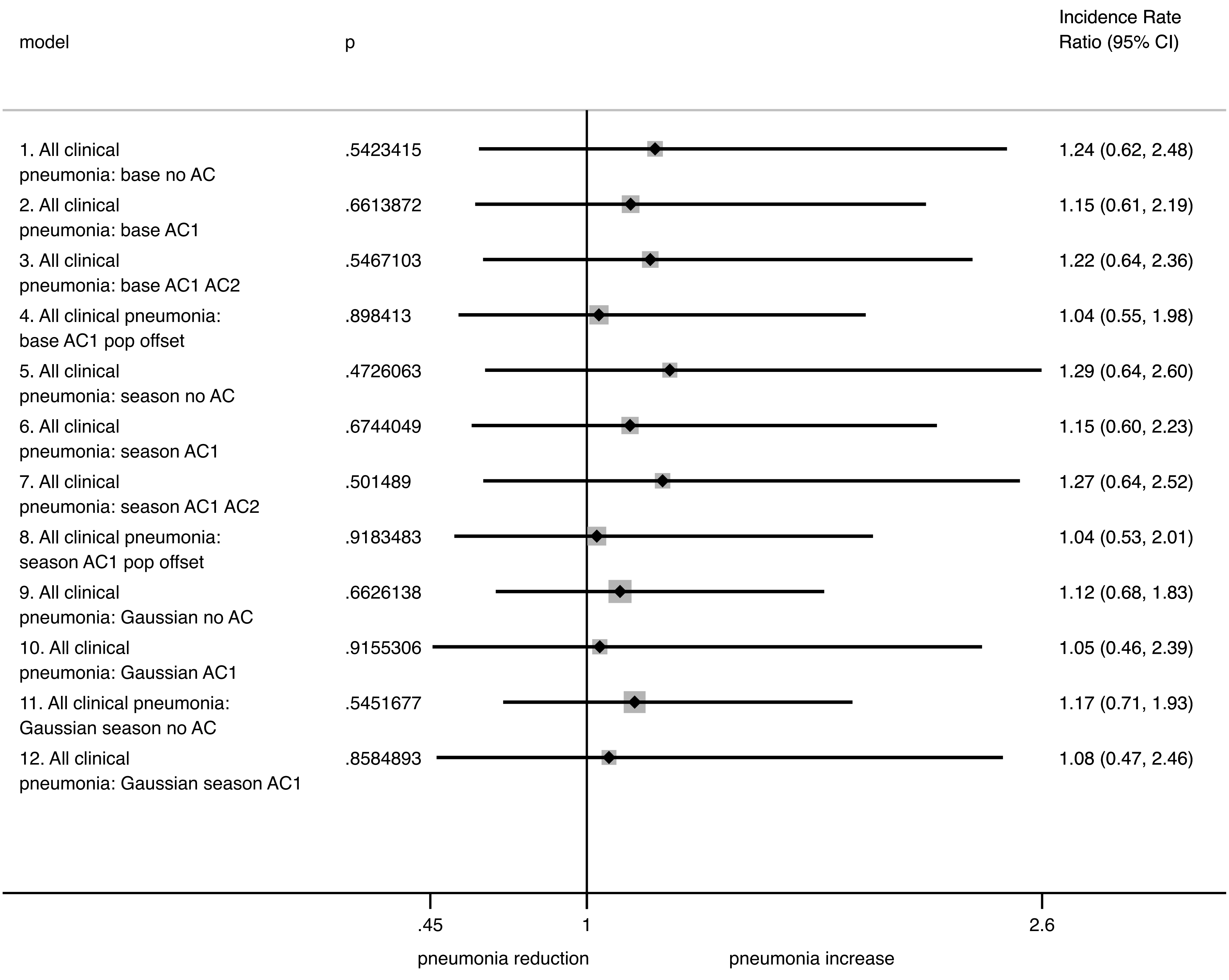

Figure A7.2 Sensitivity Analysis for Fast breathing pneumonia models - Hospitals

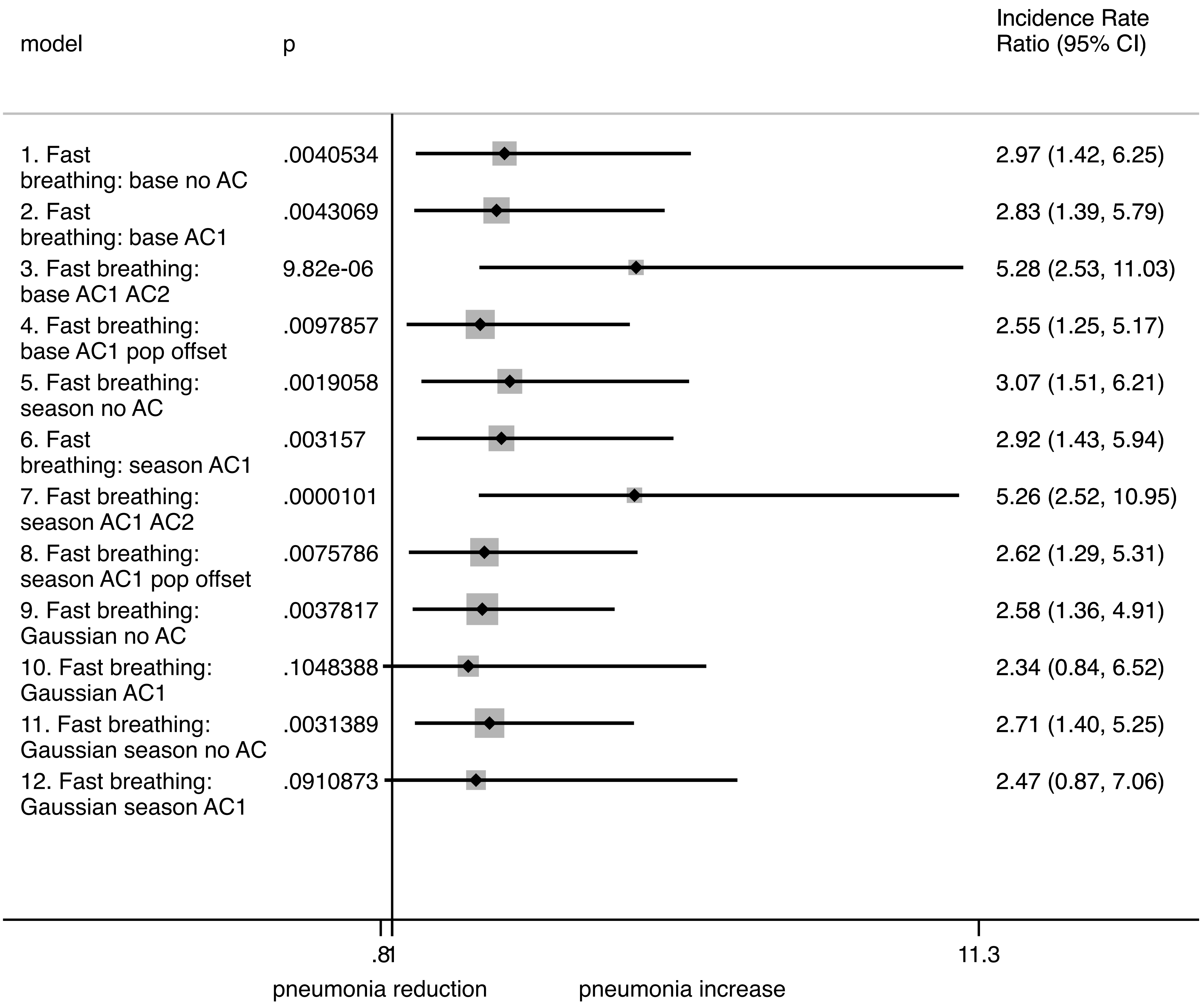

Figure A7.3 Sensitivity Analysis for Chest indrawing pneumonia models - Hospitals

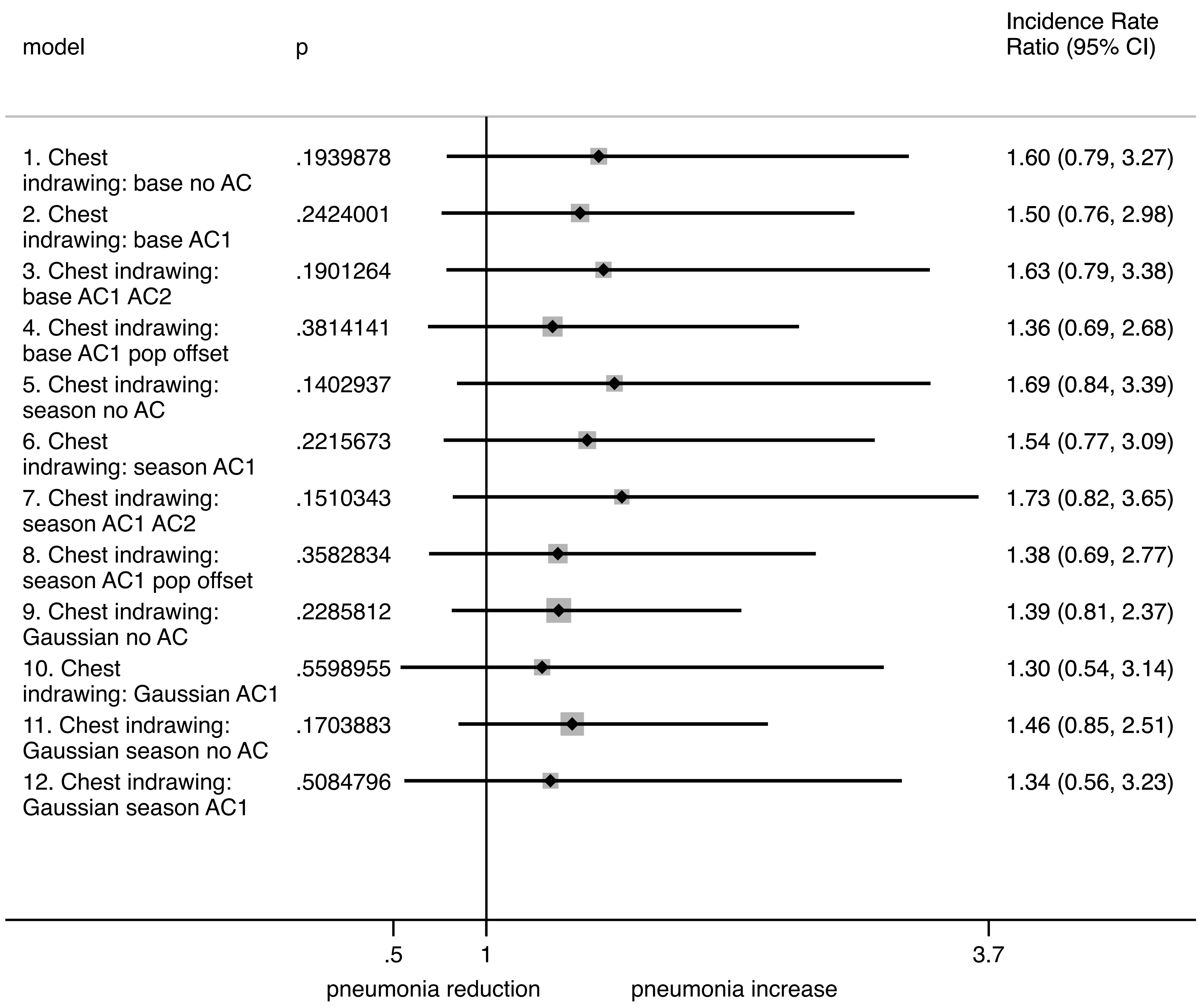

Figure A7.4 Sensitivity Analysis for Danger sign(s) pneumonia models - Hospitals

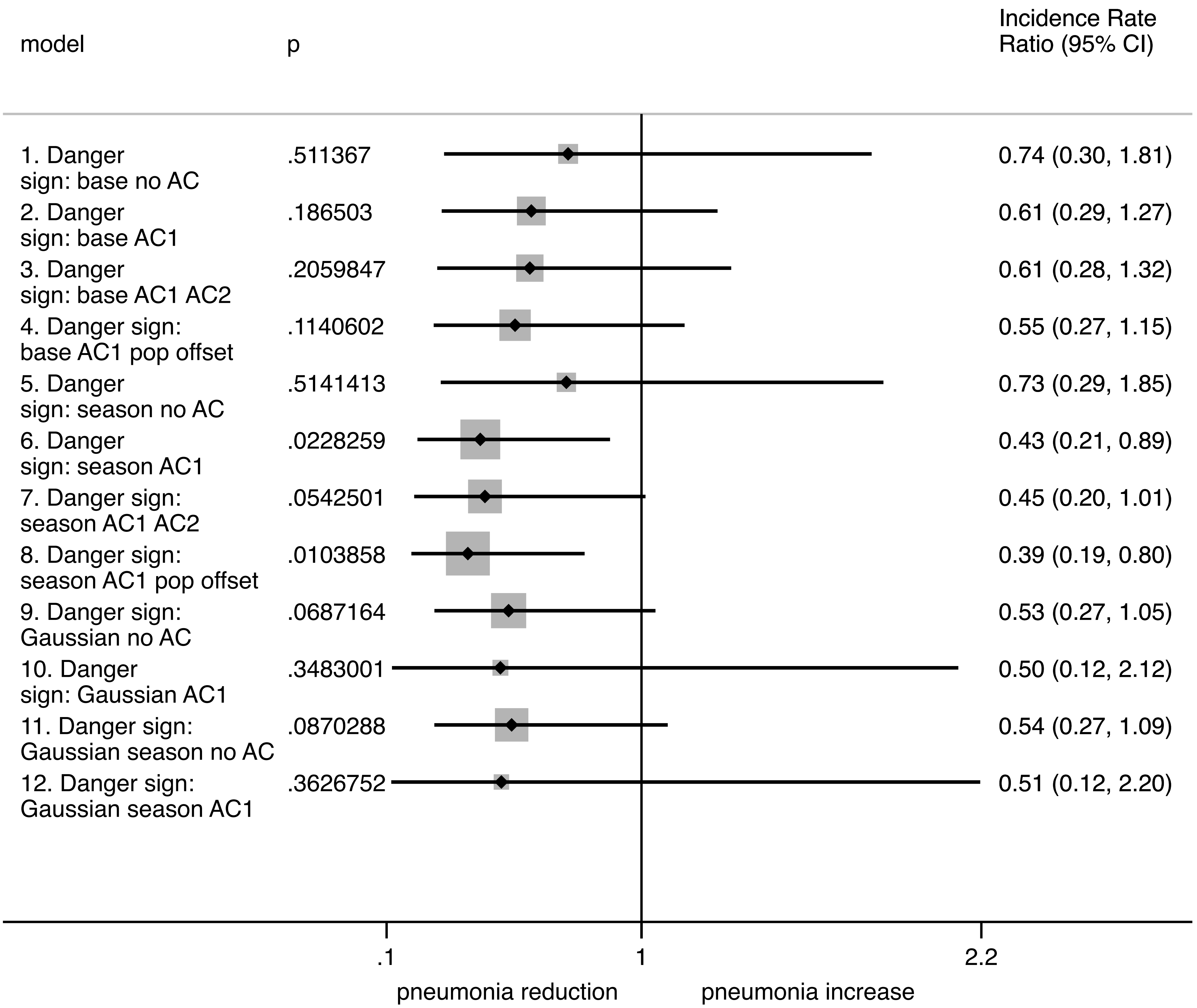

Figure A7.5 Sensitivity Analysis for Hypoxemic pneumonia models - Hospitals

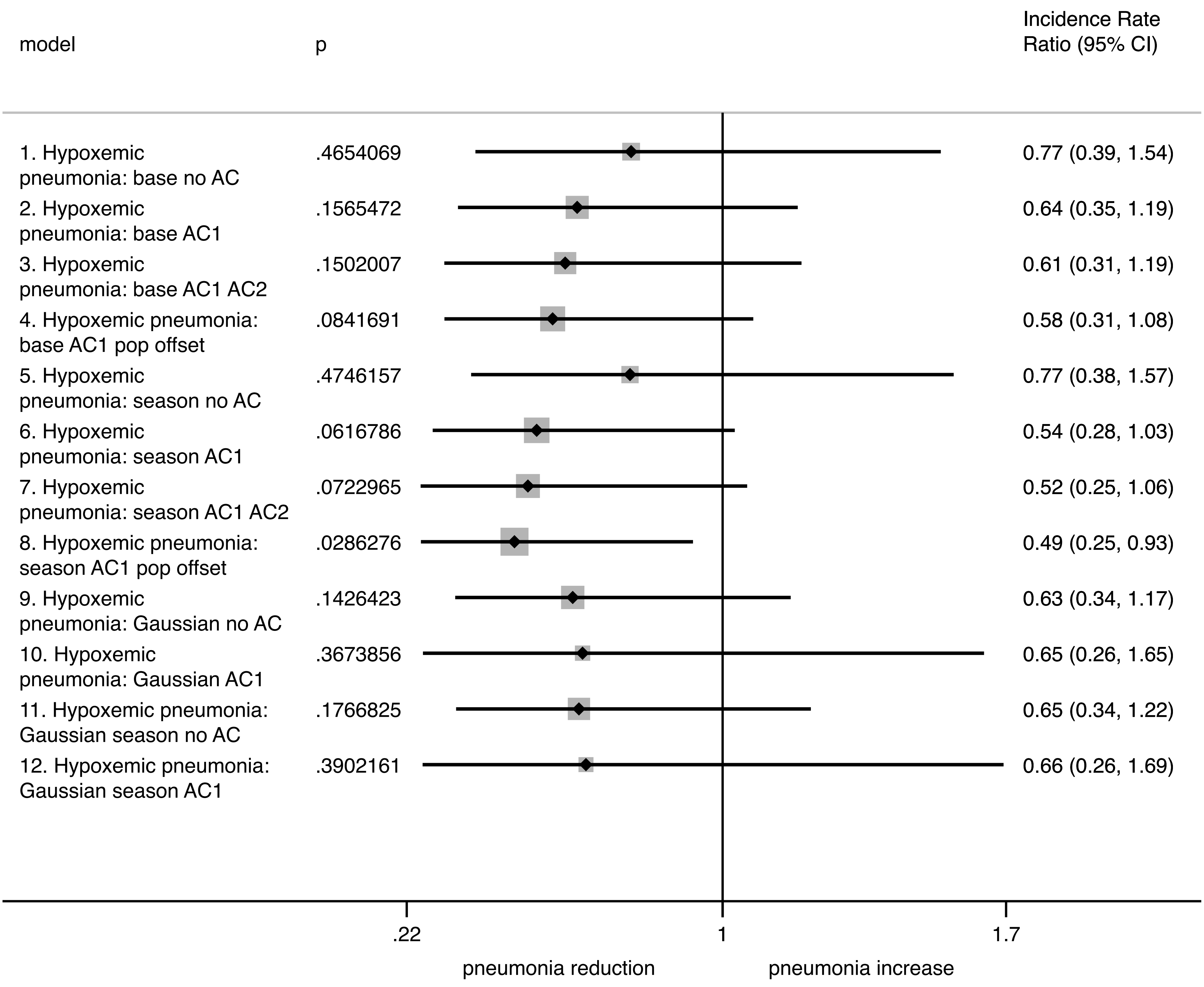

Figure A7.6 Sensitivity Analysis for Died pneumonia models - Hospitals

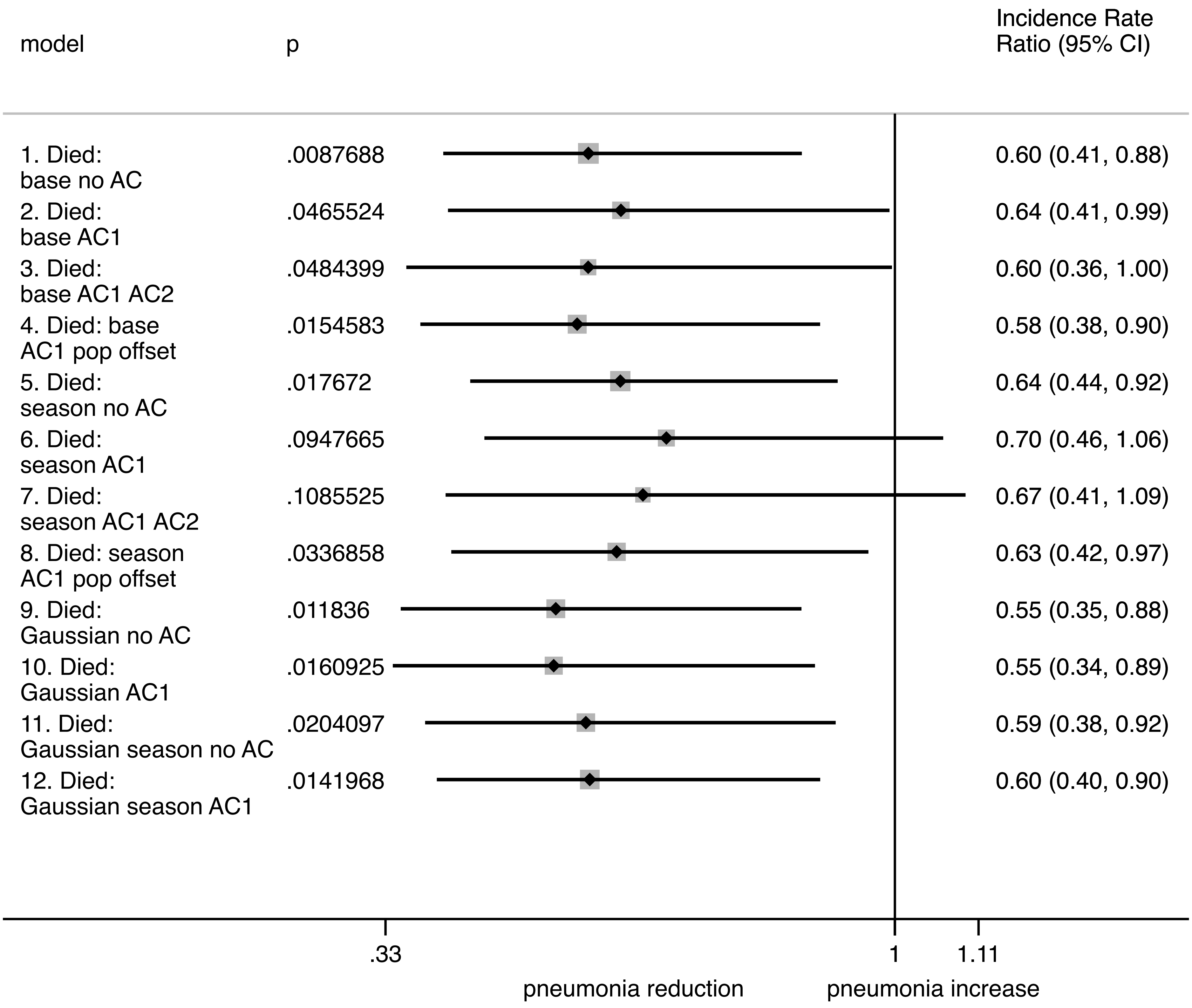

Figure A7.7

Sensitivity Analysis for proportion danger sign pneumonia models - Hospitals

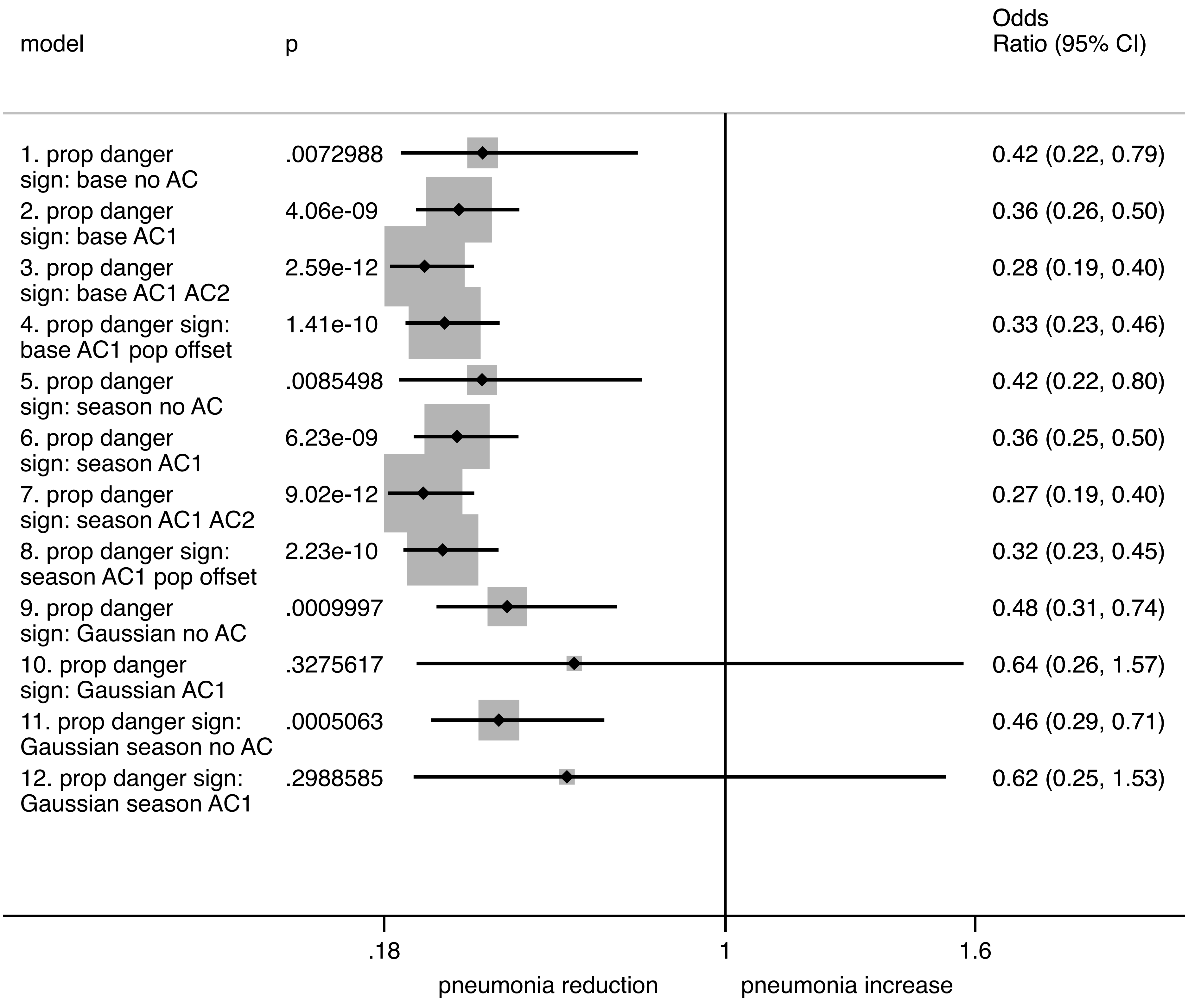

Figure A7.8 Sensitivity Analysis for All Clinical pneumonia models - Health Centres

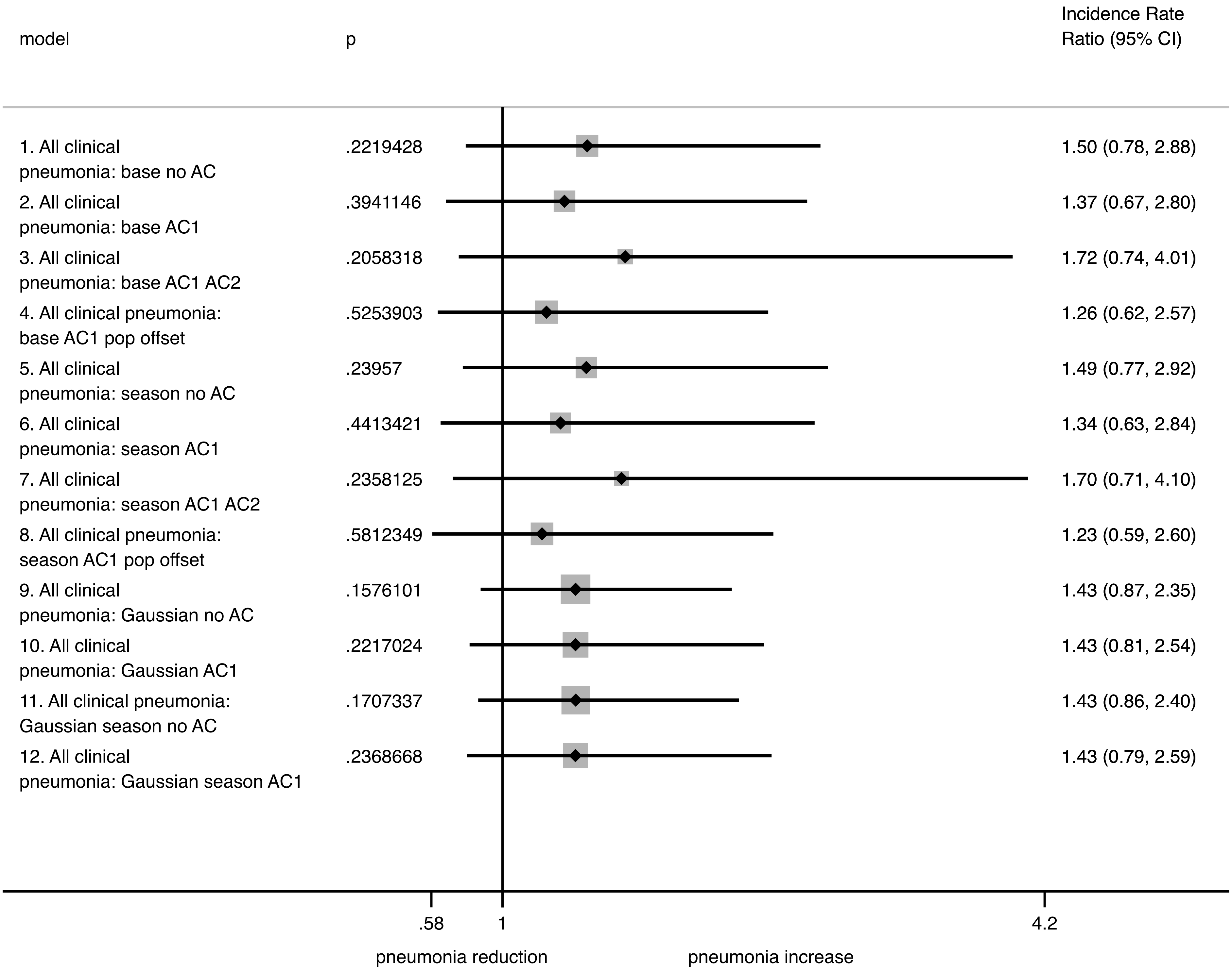

Figure A7.9 Sensitivity Analysis for Fast breathing pneumonia models - Health Centres

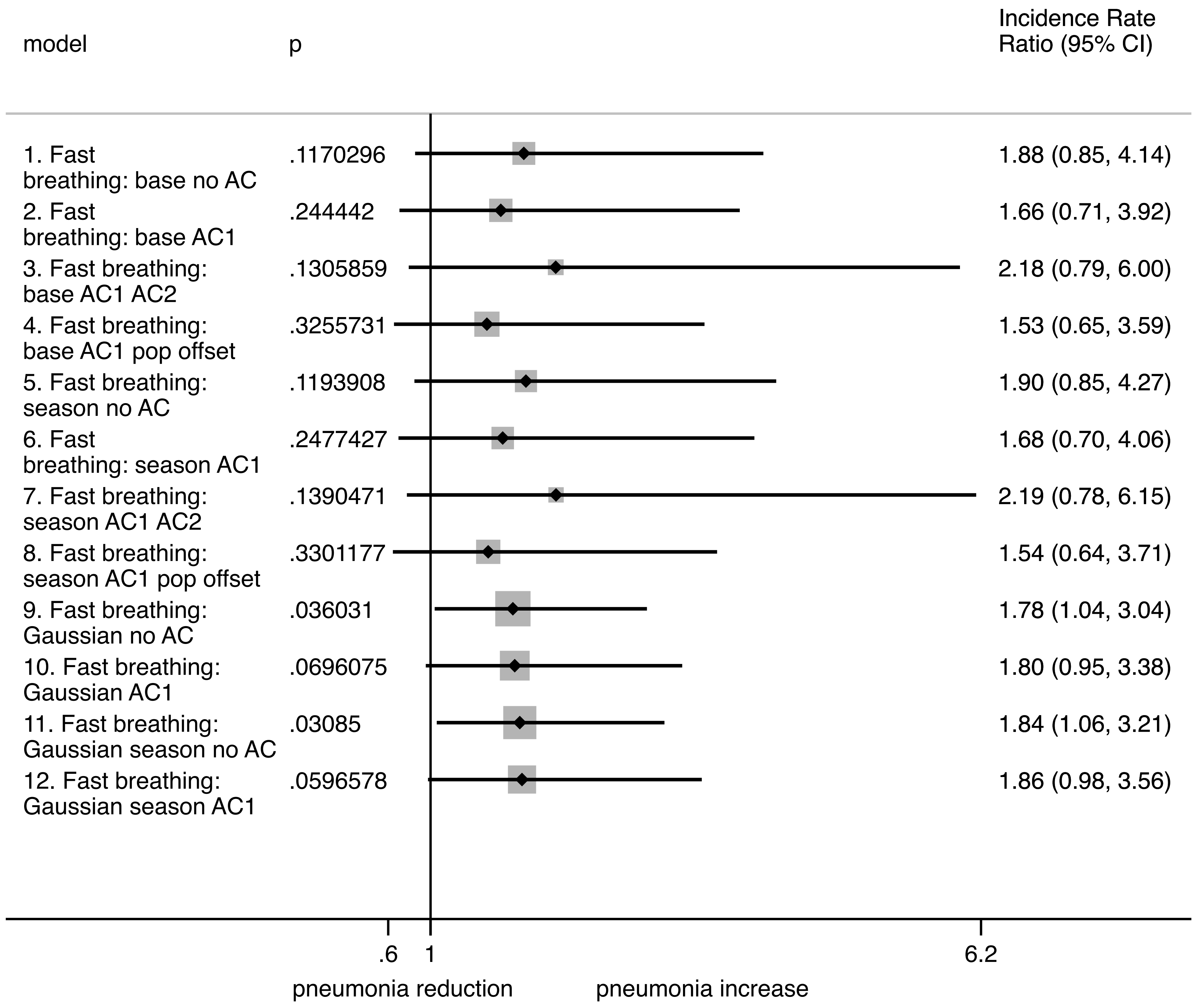

Figure A7.10

Sensitivity Analysis for Chest indrawing pneumonia models - Health Centres

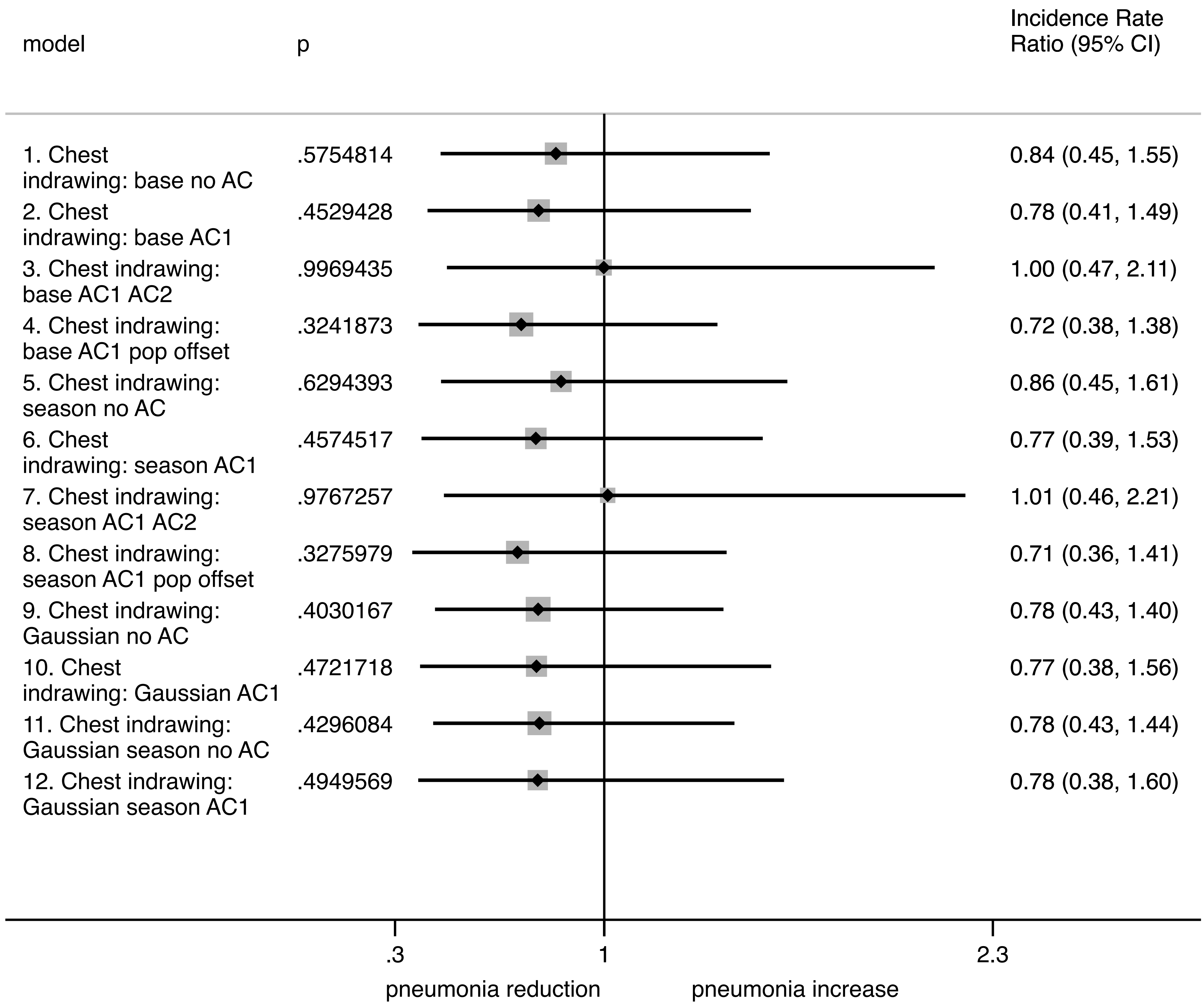

Figure A7.11

Sensitivity Analysis for Danger sign(s) pneumonia models - Health Centres

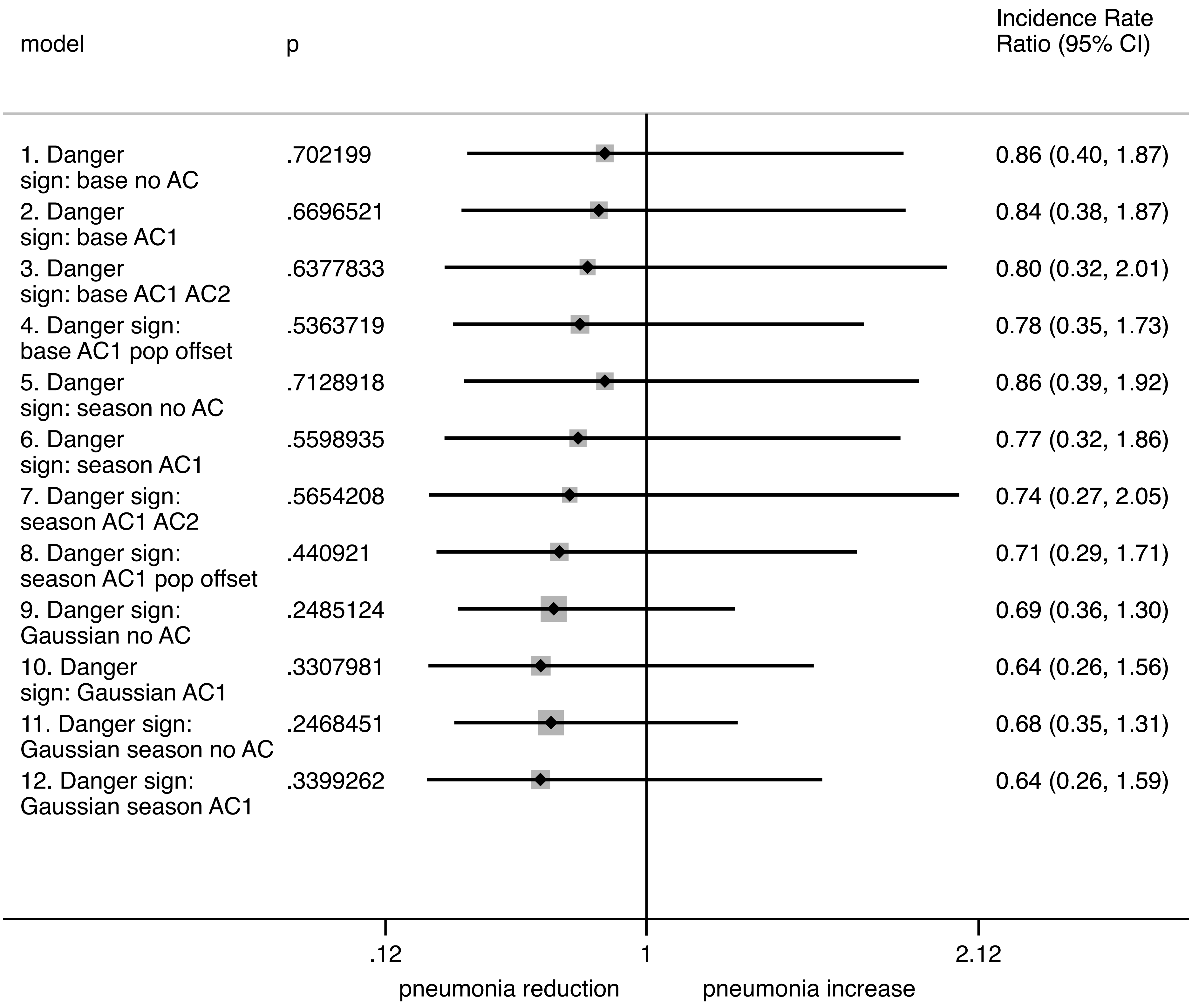

Figure A7.12

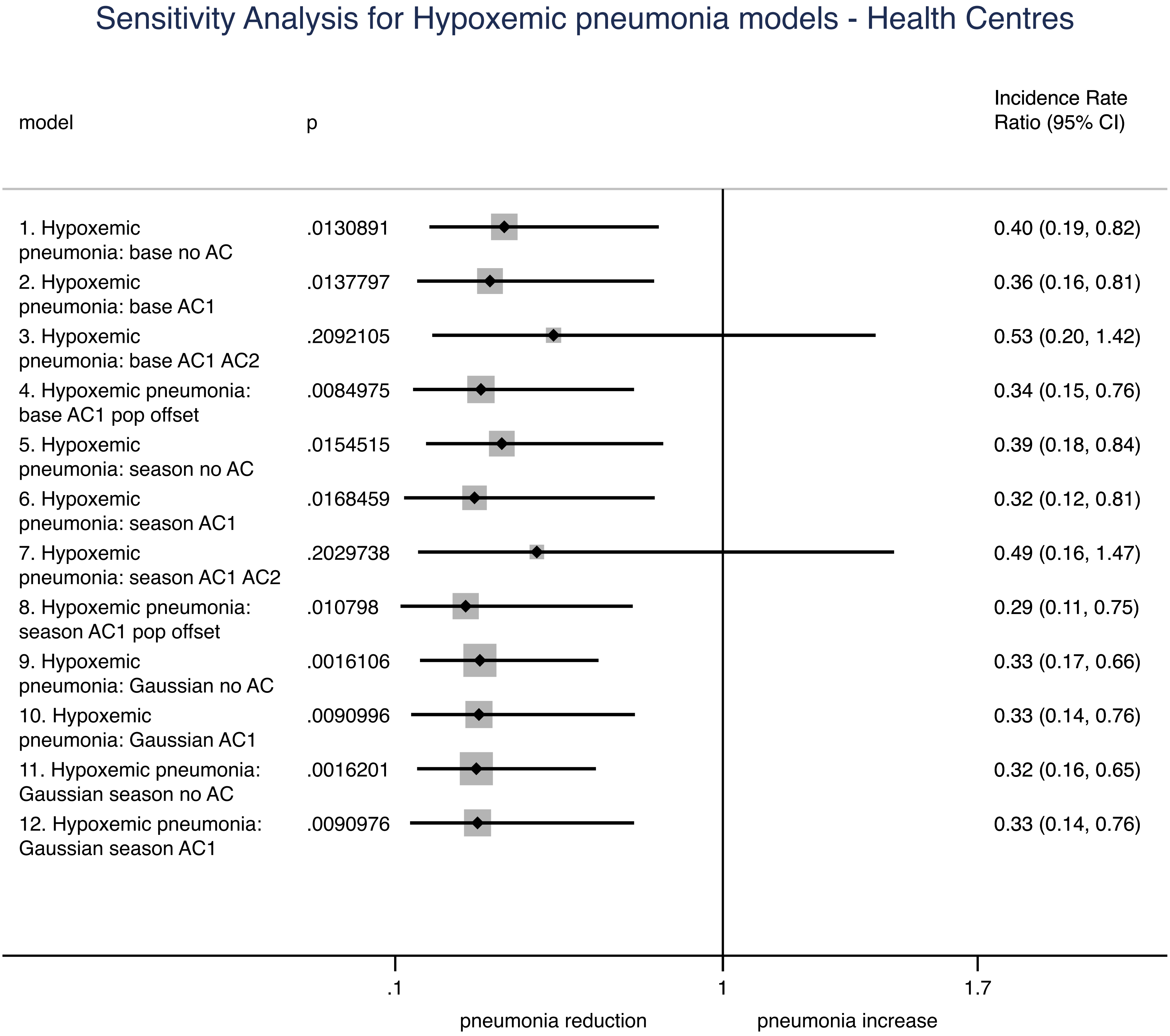
Sensitivity Analysis for Hypoxemic pneumonia models - Health Centres
model
p
Incidence Rate Ratio (95% CI)
1. Hypoxemic pneumonia: base no AC
.0130891
0.40 (0.19, 0.82)
2. Hypoxemic pneumonia: base AC1
.0137797
0.36 (0.16, 0.81)
3. Hypoxemic pneumonia: base AC1 AC2
.2092105
0.53 (0.20, 1.42)
4. Hypoxemic pneumonia: base AC1 pop offset
.0084975
0.34 (0.15, 0.76)
5. Hypoxemic pneumonia: season no AC
.0154515
0.39 (0.18, 0.84)
6. Hypoxemic pneumonia: season AC1
.0168459
0.32 (0.12, 0.81)
7. Hypoxemic pneumonia: season AC1 AC2
.2029738
0.49 (0.16, 1.47)
8. Hypoxemic pneumonia: season AC1 pop offset
.010798
0.29 (0.11, 0.75)
9. Hypoxemic pneumonia: Gaussian no AC
.0016106
0.33 (0.17, 0.66)
10. Hypoxemic pneumonia: Gaussian AC1
.0090996
0.33 (0.14, 0.76)
11. Hypoxemic pneumonia: Gaussian season no AC
.0016201
0.32 (0.16, 0.65)
12. Hypoxemic pneumonia: Gaussian season AC1
.0090976
0.33 (0.14, 0.76)
.1
1
1.7
pneumonia reduction
pneumonia increase

Figure A7.13

Sensitivity Analysis for proportion danger sign pneumonia models - Health Centres

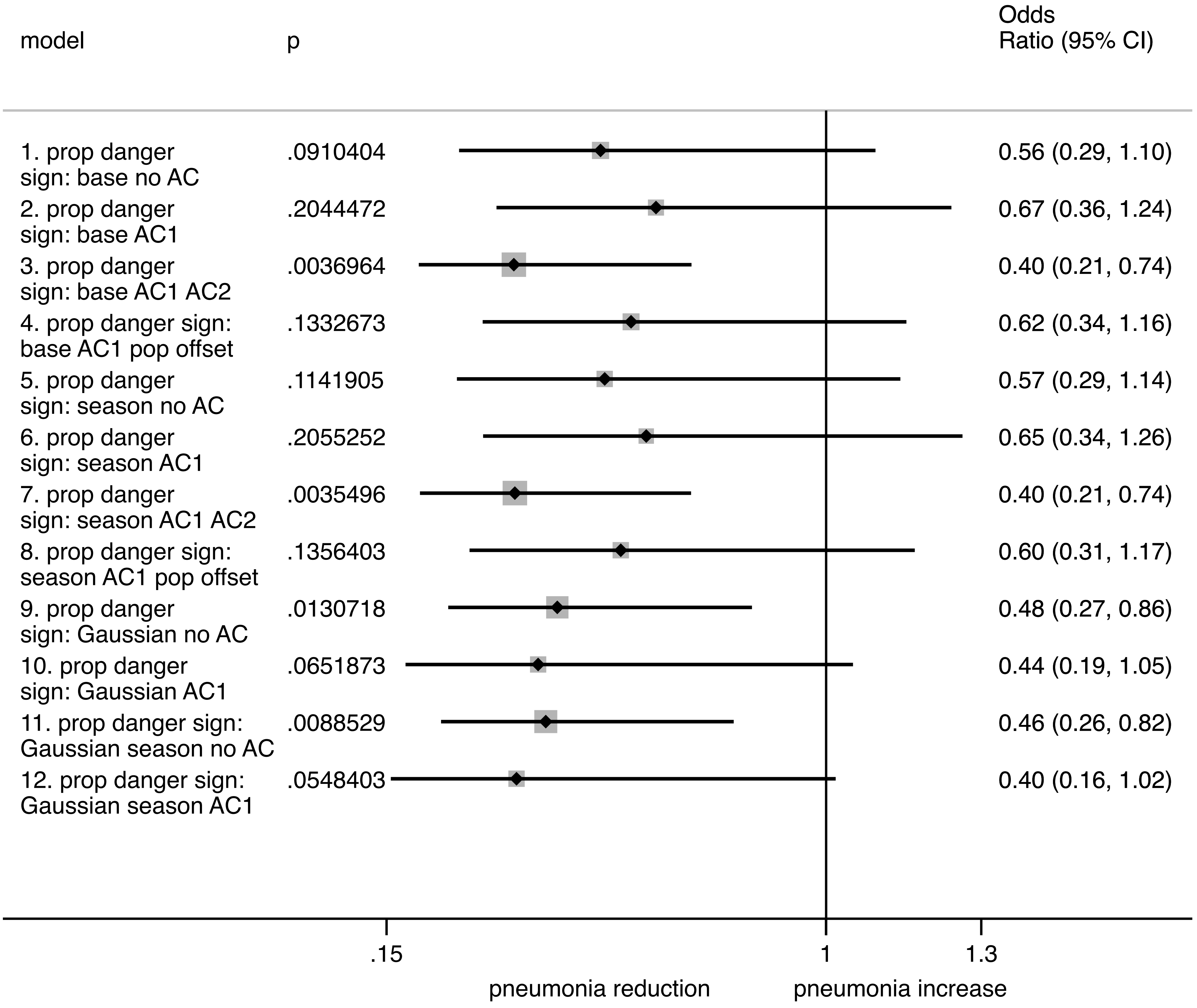

Figure A7.14

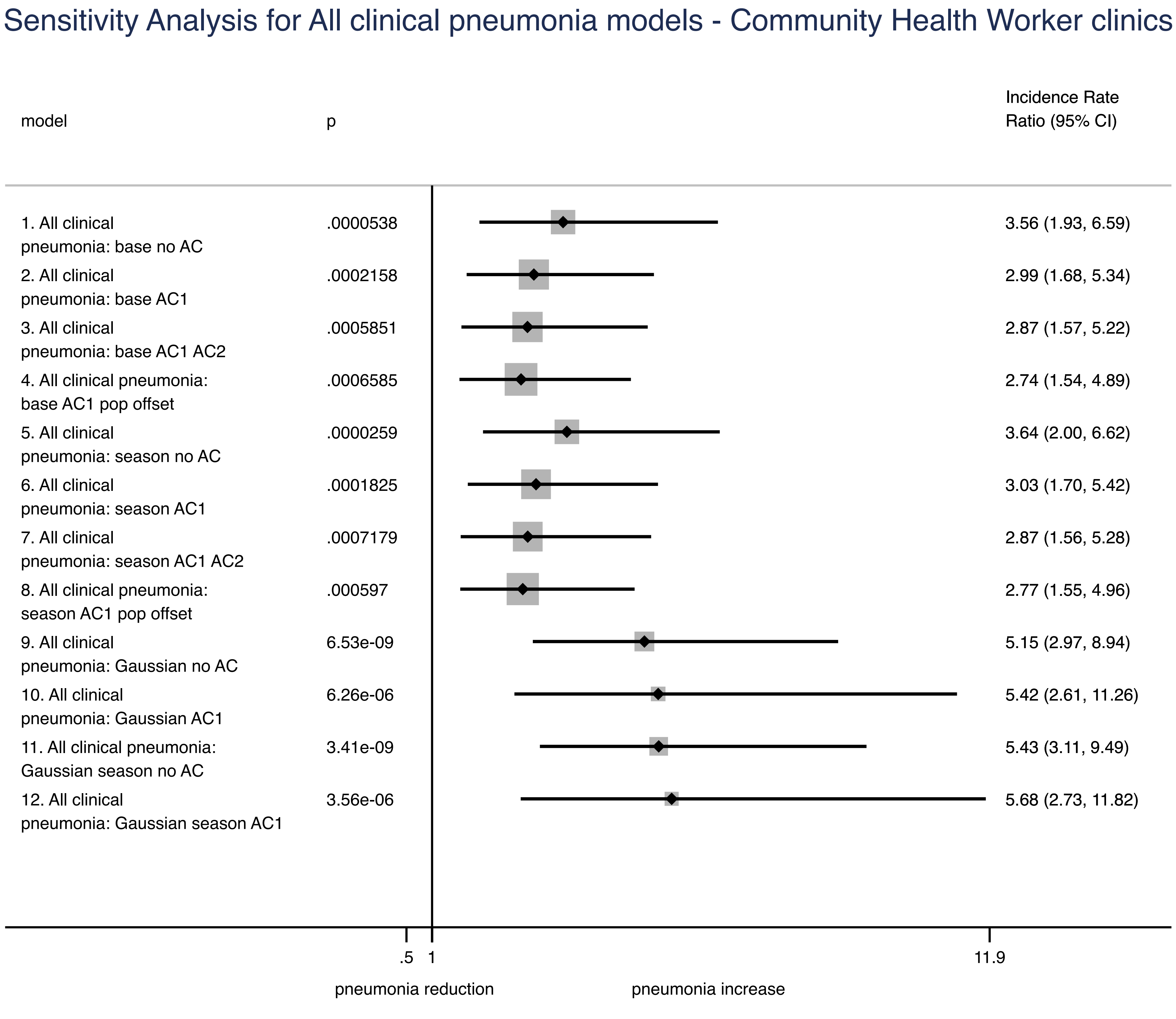
Sensitivity Analysis for All clinical pneumonia models - Community Health Worker clinics
model
p
Incidence Rate Ratio (95% CI)
1. All clinical pneumonia: base no AC
.0000538
3.56 (1.93, 6.59)
2. All clinical pneumonia: base AC1
.0002158
2.99 (1.68, 5.34)
3. All clinical pneumonia: base AC1 AC2
.0005851
2.87 (1.57, 5.22)
4. All clinical pneumonia: base AC1 pop offset
.0006585
2.74 (1.54, 4.89)
5. All clinical pneumonia: season no AC
.0000259
3.64 (2.00, 6.62)
6. All clinical pneumonia: season AC1
.0001825
3.03 (1.70, 5.42)
7. All clinical pneumonia: season AC1 AC2
.0007179
2.87 (1.56, 5.28)
8. All clinical pneumonia: season AC1 pop offset
.000597
2.77 (1.55, 4.96)
9. All clinical pneumonia: Gaussian no AC
6.53e-09
5.15 (2.97, 8.94)
10. All clinical pneumonia: Gaussian AC1
6.26e-06
5.42 (2.61, 11.26)
11. All clinical pneumonia: Gaussian season no AC
3.41e-09
5.43 (3.11, 9.49)
12. All clinical pneumonia: Gaussian season AC1
3.56e-06
5.68 (2.73, 11.82)
.5
1
11.9
pneumonia reduction
pneumonia increase

Figure A7.15

Sensitivity Analysis for Fast breathing pneumonia models - Community Health Worker clinics

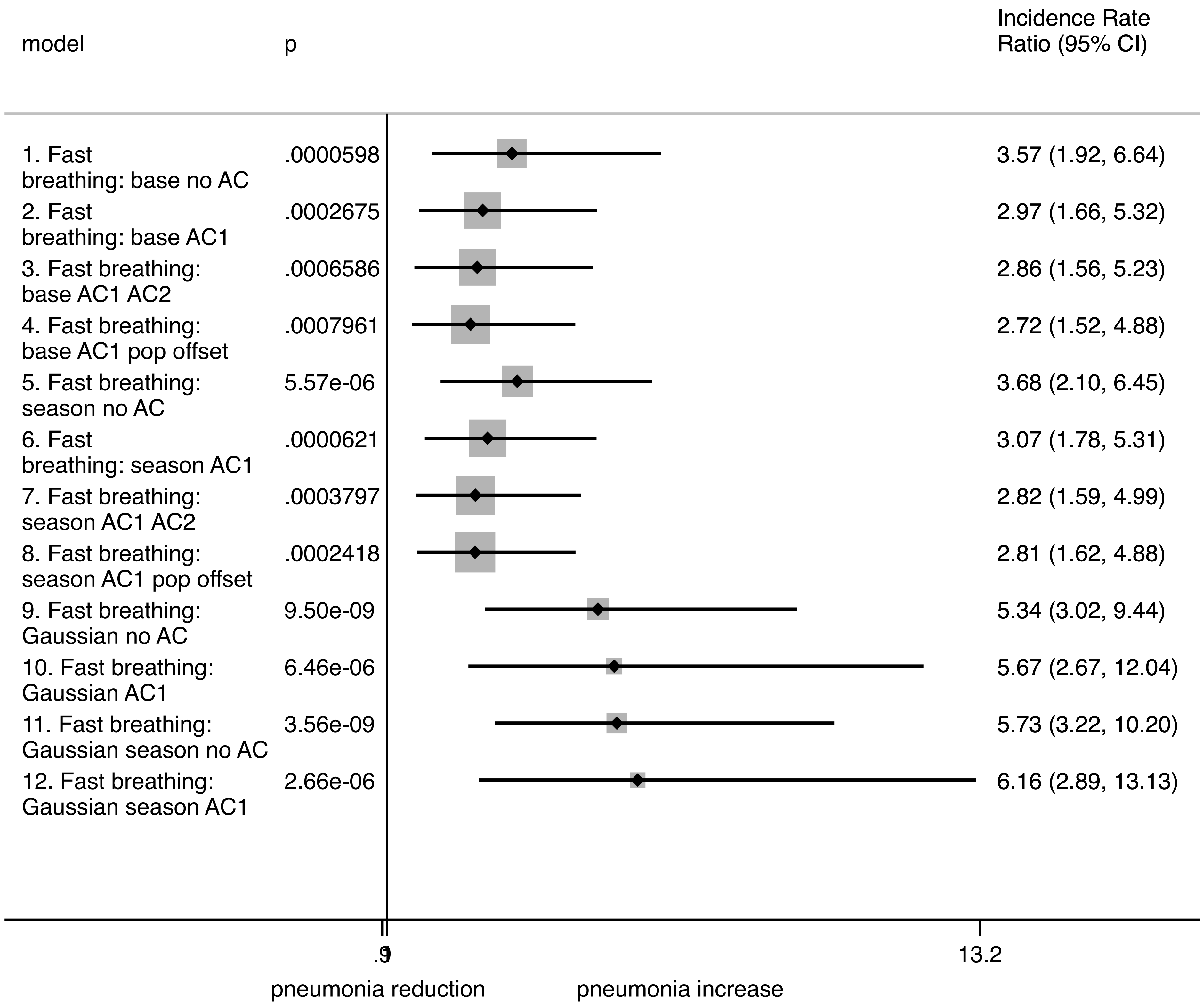

Figure A7.16

Sensitivity Analysis for Chest indrawing pneumonia models - Community Health Worker clinics

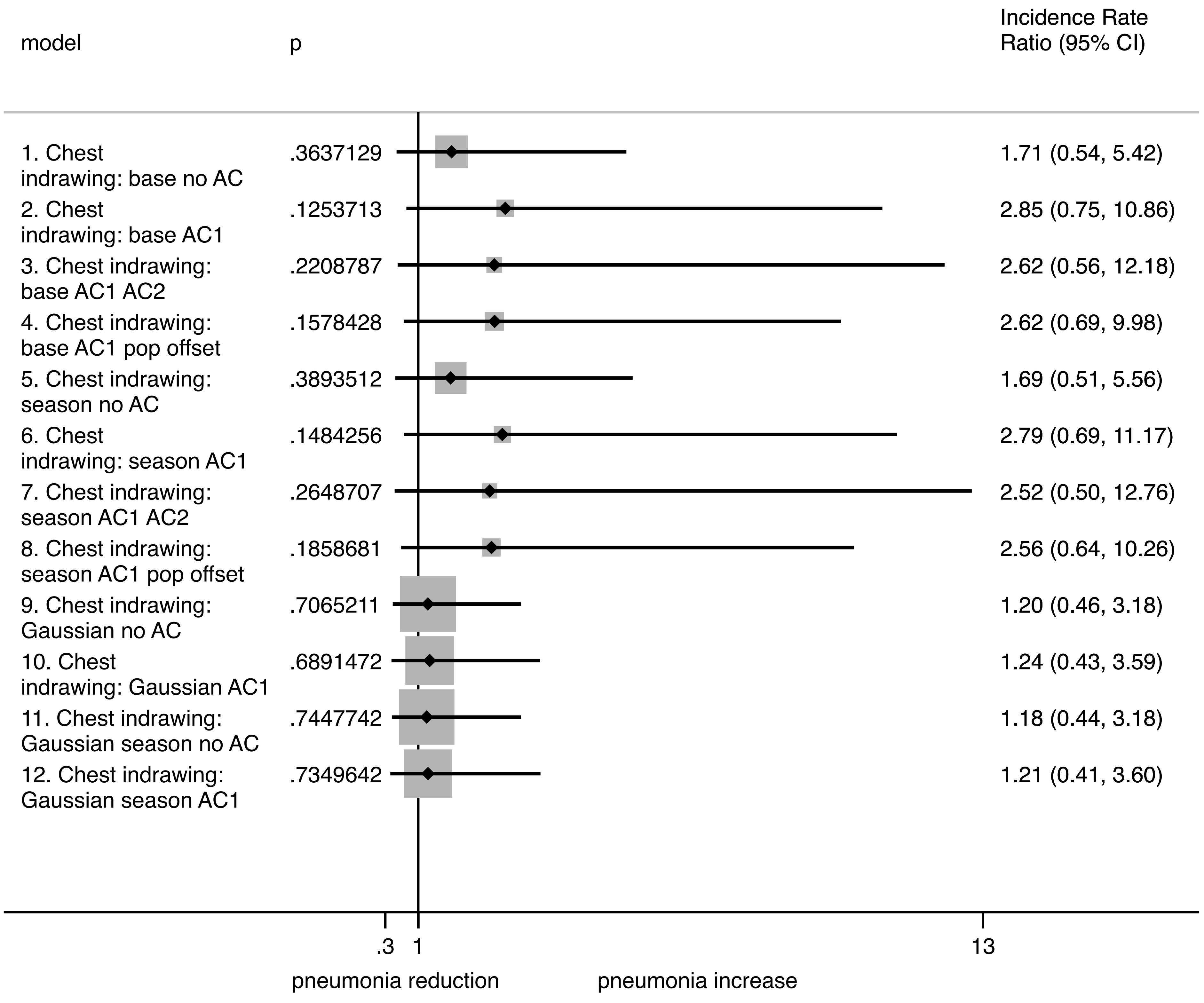

Figure A7.17

Sensitivity Analysis for Danger sign(s) pneumonia models - Community Health Worker clinics

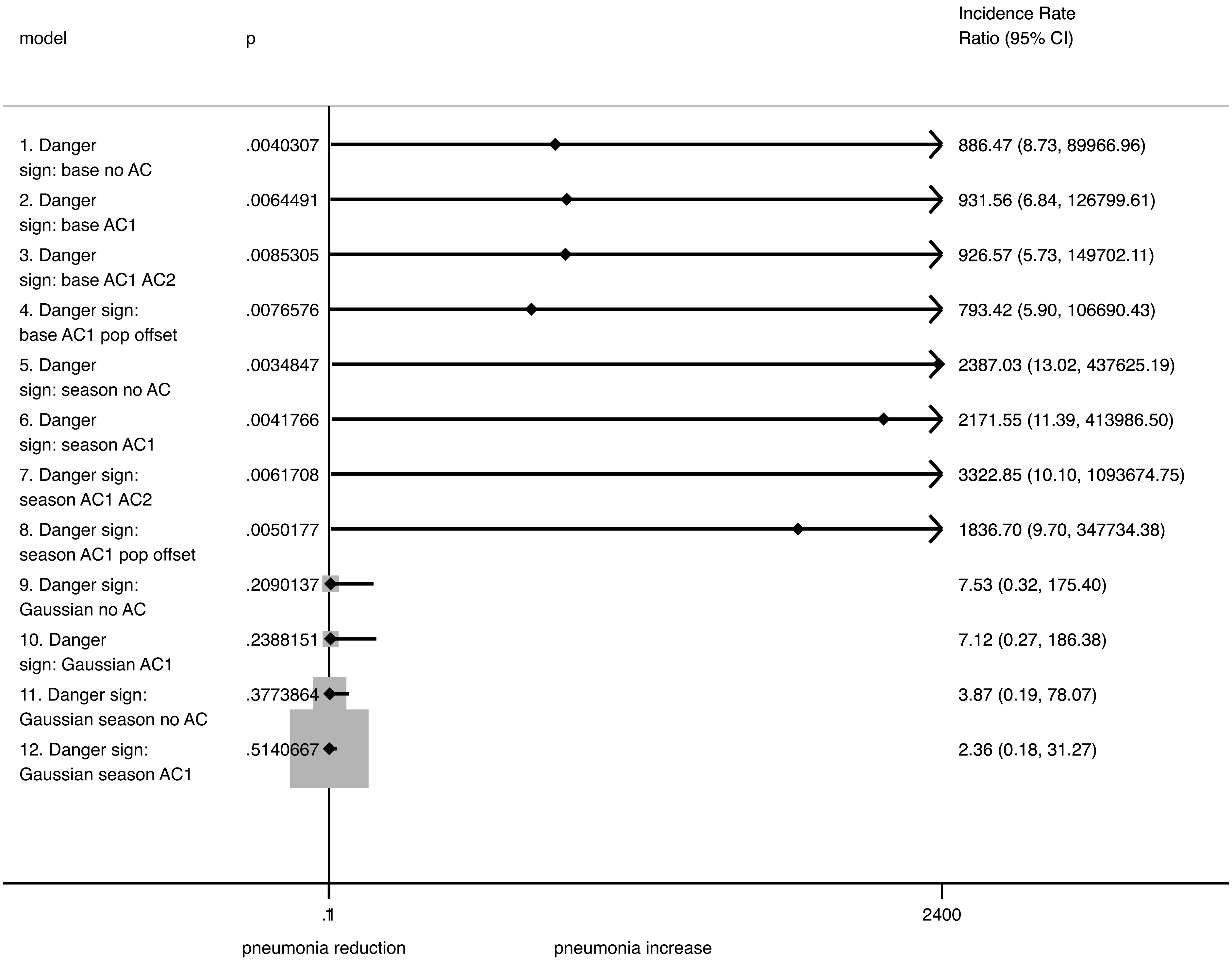

Figure A7.18

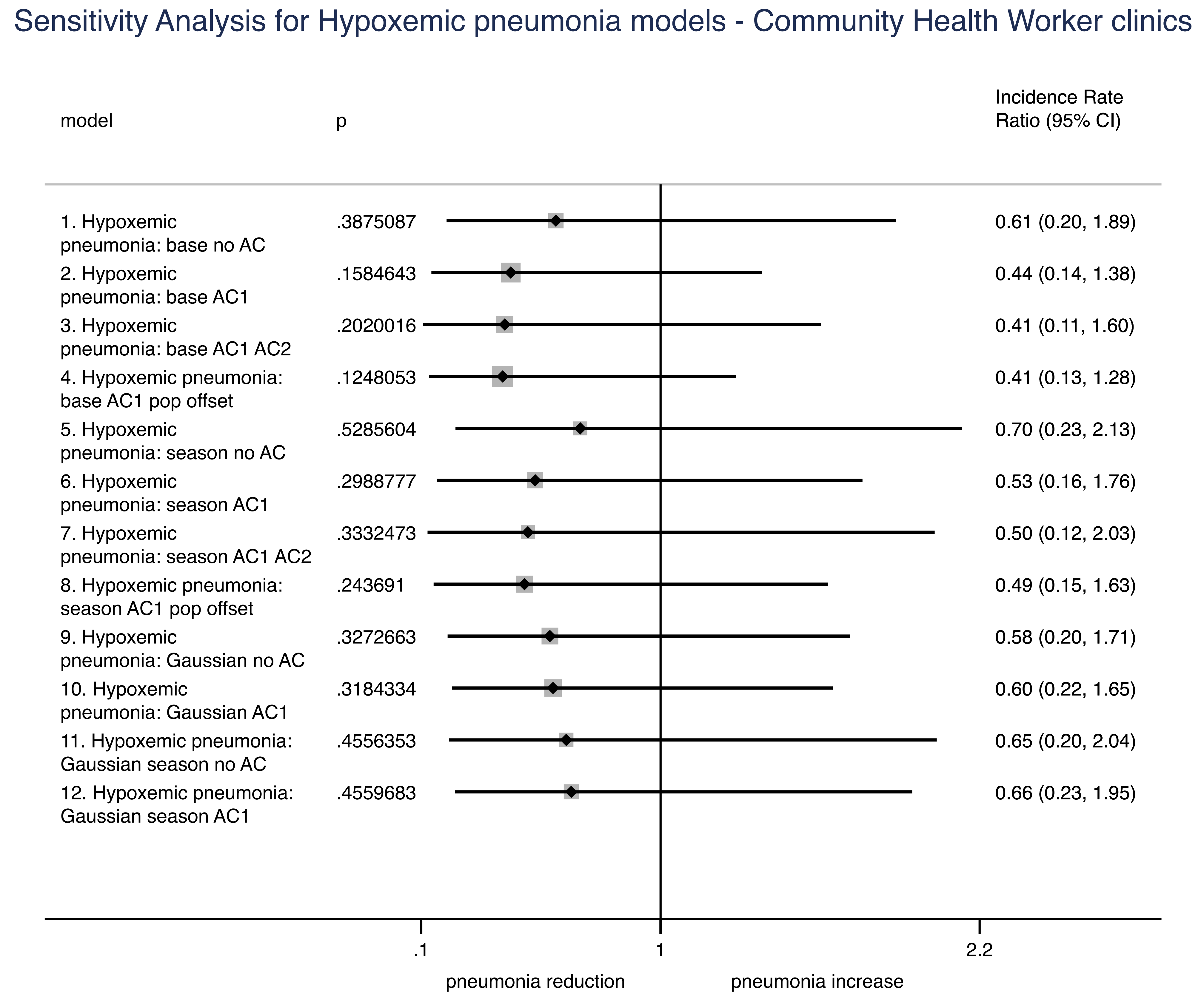

Sensitivity Analysis for Hypoxemic pneumonia models - Community Health Worker clinics
model
p
Incidence Rate Ratio (95% CI)
1. Hypoxemic pneumonia: base no AC
.3875087
0.61 (0.20, 1.89)
2. Hypoxemic pneumonia: base AC1
.1584643
0.44 (0.14, 1.38)
3. Hypoxemic pneumonia: base AC1 AC2
.2020016
0.41 (0.11, 1.60)
4. Hypoxemic pneumonia: base AC1 pop offset
.1248053
0.41 (0.13, 1.28)
5. Hypoxemic pneumonia: season no AC
.5285604
0.70 (0.23, 2.13)
6. Hypoxemic pneumonia: season AC1
.2988777
0.53 (0.16, 1.76)
7. Hypoxemic pneumonia: season AC1 AC2
.3332473
0.50 (0.12, 2.03)
8. Hypoxemic pneumonia: season AC1 pop offset
.243691
0.49 (0.15, 1.63)
9. Hypoxemic pneumonia: Gaussian no AC
.3272663
0.58 (0.20, 1.71)
10. Hypoxemic pneumonia: Gaussian AC1
.3184334
0.60 (0.22, 1.65)
11. Hypoxemic pneumonia: Gaussian season no AC
.4556353
0.65 (0.20, 2.04)
12. Hypoxemic pneumonia: Gaussian season AC1
.4559683
0.66 (0.23, 1.95)
.1
1
2.2
pneumonia reduction
pneumonia increase

Figure A7.19

Sensitivity Analysis for proportion danger sign pneumonia models - Community Health Worker clinics

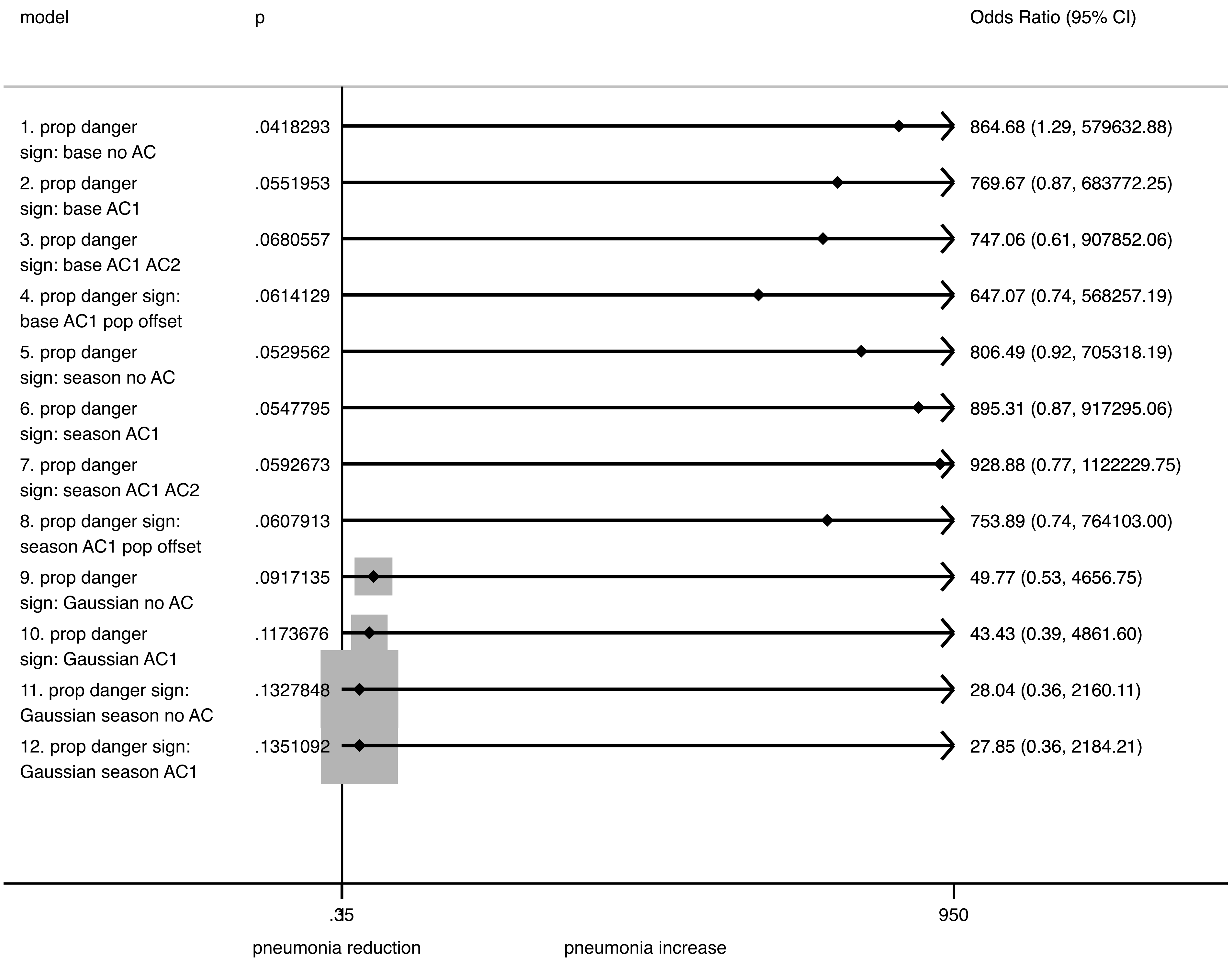

Supplement: S7 Appendix — (PDF) [file pone.0168209.s007.pdf]
