# Supplementary material for: Impact of the 13-Valent Pneumococcal Conjugate Vaccine on Clinical and Hypoxemic Childhood Pneumonia over Three Years in Central Malawi: An Observational Study
Source: PLoS One. 2017 Jan 4;12(1):e0168209. doi: 10.1371/journal.pone.0168209 (PMC5215454; doi:10.1371/journal.pone.0168209)

Appendix 8: Time-Series Regression Results with Age Category as an Effect Modifier

Table A8: Age Category as an Effect Modifier: Time-series regression results with the association of 76% three-dose PCV13 coverage on pneumonia outcomes highlighted

Model and covariate [a]	All clinical pneumonia [a] IRR (95%CI); p-value
*agecat*3dose* (B_1)	
0-5 months	1.027 (0.891, 1.500) p=0.891
6-23 months	2.328 (1.652, 3.282) p<0.0001
24-59 months	1.028 (0.706, 1.497); p=0.886
L1.dres (B_3)	1.028 (1.018 1.038); p<0.0001
constant (B_0)	236.4 (182.0, 307.2); p<0.0001

Model and covariate [a]	Fast breathing pneumonia [a] IRR (95%CI); p-value	Chest indrawing pneumonia IRR (95%CI); p-value	Danger sign pneumonia [a] IRR (95%CI); p-value	Hypoxemic pneumonia [a] IRR (95%CI); p-value	Mortality [a] IRR (95%CI); p-value	Proportion danger sign pneumonia [a] OR (95%CI); p-value
*agecat*3dose* (B_1)						
0-5 months	0.882 (0.592, 1.316) p=0.540	1.268 (0.813, 1.979) p=0.295	0.685 (0.4112, 1.141) p=0.146	0.567 (0.363, 0.888) p=0.013	0.741 (0.454, 1.211) p=0.232	0.655 (0.481, 0.892) p=0.007
6-23 months	3.689 (2.637, 5.162) p<0.0001	2.172 (1.435, 3.289) p<0.0001	1.021 (0.630, 1.655) p=0.932	0.848 (0.558, 1.290) p=0.442	0.920 (0.572, 1.480) p=0.732	0.348 (0.260, 0.466) p<0.0001
24-59 months	2.271 (1.597, 3.228); p<0.0001	0.719 (0.446, 1.160); p=0.177	0.227 (0.122, 0.420) p<0.0001	0.171 (0.097, 0.300); p<0.0001	0.233 (0.125, 0.433); p<0.0001	0.166 (0.116, 0.235); p<0.0001
L1.dres (B_3)	1.024 (1.007 1.042); p=0.007	1.045 (1.030, 1.061); p=0<0001	1.064 (1.049, 1.079); p<0.0001	1.091 (1.060, 1.124); p<0.0001	0.999 (0.902, 1.107); p=0.990	1.098 (1.078, 1.119); p<0.0001
constant (B_0)	76.24 (58.47, 99.41); p<0.0001	86.56 (63.1, 118.67); p<0.0001	83.65 (59.44, 117.71); p<0.0001	38.24 (28.55, 51.22); p<0.0001	7.43 (5.32, 10.37); p<0.0001	0.511 (0.414, 0.630); p<0.0001

IRR = Incidence Rate Ratio OR = Odds Ratio

[a] Observations = 87; these are the 30 calendar months in time (January 2012 to June 2014) minus one due to the inclusion of the one-month lagged residual term *L1.dres* multiplied by three for each of the age categories: 0-5 months, 6-23 months, 24-59 months

Figure A8 Age Category as an Effect Modifier: Association of 76% three-dose PCV13 coverage on pneumonia outcomes

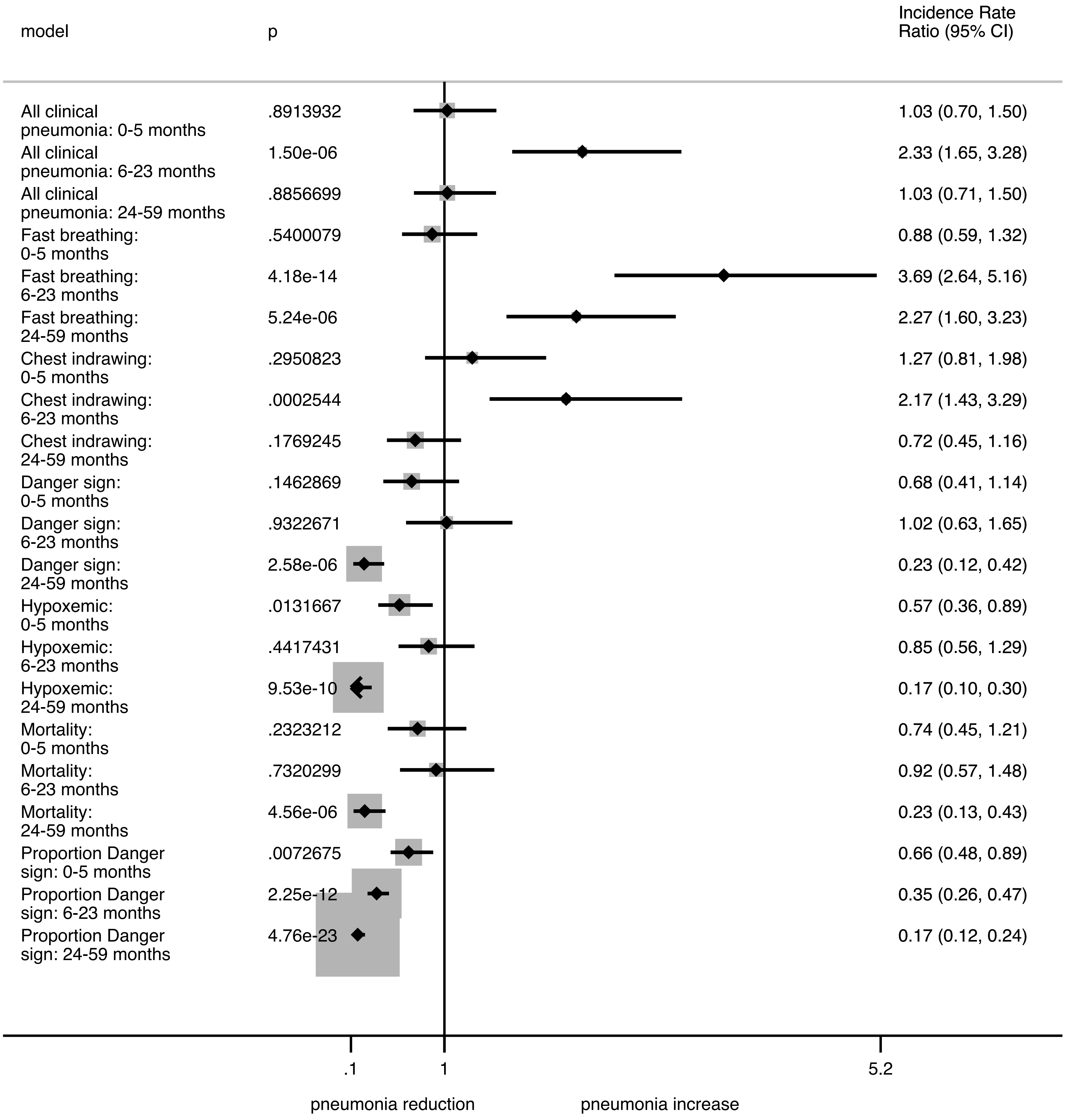

Supplement: S8 Appendix — (PDF) [file pone.0168209.s008.pdf]
