# Supplementary material for: Impact of the 13-Valent Pneumococcal Conjugate Vaccine on Clinical and Hypoxemic Childhood Pneumonia over Three Years in Central Malawi: An Observational Study
Source: PLoS One. 2017 Jan 4;12(1):e0168209. doi: 10.1371/journal.pone.0168209 (PMC5215454; doi:10.1371/journal.pone.0168209)
Supplement: S1 Checklist — (DOC) [file pone.0168209.s009.doc]

STROBE Statement—checklist of items that should be included in reports of observational studies

|  | Item No | Recommendation |
| --- | --- | --- |
| **Title and abstract** | 1 | (*a*) Indicate the study’s design with a commonly used term in the title or the abstract DONE – see Title, and Abstract lines 54-55 and 63-65 |
|  |  | (*b*) Provide in the abstract an informative and balanced summary of what was done and what was found DONE – see Abstract Methods and Findings |
| Introduction | | |
| Background/rationale | 2 | Explain the scientific background and rationale for the investigation being reported DONE – see Introduction lines 83-107 |
| Objectives | 3 | State specific objectives, including any prespecified hypotheses DONE – see Introduction lines 109-119 |
| Methods | | |
| Study design | 4 | Present key elements of study design early in the paper DONE – see lines 122-136 |
| Setting | 5 | Describe the setting, locations, and relevant dates, including periods of recruitment, exposure, follow-up, and data collection DONE – see lines 122-201 |
| Participants | 6 | (*a*) *Cohort study*—Give the eligibility criteria, and the sources and methods of selection of participants. Describe methods of follow-up DONE – see lines 54-60 and 122-230  *Case-control study*—Give the eligibility criteria, and the sources and methods of case ascertainment and control selection. Give the rationale for the choice of cases and controls  *Cross-sectional study*—Give the eligibility criteria, and the sources and methods of selection of participants |
|  |  | (*b*) *Cohort study*—For matched studies, give matching criteria and number of exposed and unexposed  *Case-control study*—For matched studies, give matching criteria and the number of controls per case |
| Variables | 7 | Clearly define all outcomes, exposures, predictors, potential confounders, and effect modifiers. Give diagnostic criteria, if applicable DONE – see lines 232-295 |
| Data sources/ measurement | 8* | For each variable of interest, give sources of data and details of methods of assessment (measurement). Describe comparability of assessment methods if there is more than one group |
| Bias | 9 | Describe any efforts to address potential sources of bias DONE – see lines 233-274, 493-502 and 561-587 |
| Study size | 10 | Explain how the study size was arrived at DONE – see lines 123-129 |
| Quantitative variables | 11 | Explain how quantitative variables were handled in the analyses. If applicable, describe which groupings were chosen and why DONE – see lines 232-295 |
| Statistical methods | 12 | (*a*) Describe all statistical methods, including those used to control for confounding DONE – see lines 232-295 |
|  |  | (*b*) Describe any methods used to examine subgroups and interactions DONE – see lines 252-264 and 276-287 |
|  |  | (*c*) Explain how missing data were addressed DONE – see lines 286-287 |
|  |  | (*d*) *Cohort study*—If applicable, explain how loss to follow-up was addressed  *Case-control study*—If applicable, explain how matching of cases and controls was addressed  *Cross-sectional study*—If applicable, describe analytical methods taking account of sampling strategy |
|  |  | (*e*) Describe any sensitivity analyses DONE – see lines 293-295 |

| Results | | |
| --- | --- | --- |
| Participants | 13* | (a) Report numbers of individuals at each stage of study—eg numbers potentially eligible, examined for eligibility, confirmed eligible, included in the study, completing follow-up, and analysed DONE – see lines 298-336 including Table 1 (also Appendix 4) |
|  |  | (b) Give reasons for non-participation at each stage |
|  |  | (c) Consider use of a flow diagram |
| Descriptive data | 14* | (a) Give characteristics of study participants (eg demographic, clinical, social) and information on exposures and potential confounders DONE – see Table 1 (also Appendix 4) |
|  |  | (b) Indicate number of participants with missing data for each variable of interest DONE – see Table 1 (also Appendix 4) |
|  |  | (c) *Cohort study*—Summarise follow-up time (eg, average and total amount) DONE – see line 298 |
| Outcome data | 15* | *Cohort study*—Report numbers of outcome events or summary measures over time DONE – see Figure 2, Tables 1-3 and Results section (also appendices 4-8) |
|  |  | *Case-control study—*Report numbers in each exposure category, or summary measures of exposure |
|  |  | *Cross-sectional study—*Report numbers of outcome events or summary measures |
| Main results | 16 | (*a*) Give unadjusted estimates and, if applicable, confounder-adjusted estimates and their precision (eg, 95% confidence interval). Make clear which confounders were adjusted for and why they were included DONE – see lines 338-437 including Tables 2 and 3 (also appendices 4-8) |
|  |  | (*b*) Report category boundaries when continuous variables were categorized DONE – see Appendices 4 and 8 |
|  |  | (*c*) If relevant, consider translating estimates of relative risk into absolute risk for a meaningful time period DONE – see lines 451-456 |
| Other analyses | 17 | Report other analyses done—eg analyses of subgroups and interactions, and sensitivity analyses DONE – see Appendices 5-8 |
| Discussion | | |
| Key results | 18 | Summarise key results with reference to study objectives DONE – see lines 441-461 |
| Limitations | 19 | Discuss limitations of the study, taking into account sources of potential bias or imprecision. Discuss both direction and magnitude of any potential bias DONE – see lines 463-530, 561-587 |
| Interpretation | 20 | Give a cautious overall interpretation of results considering objectives, limitations, multiplicity of analyses, results from similar studies, and other relevant evidence DONE – see Discussion, and concluding paragraph: lines 589-601 |
| Generalisability | 21 | Discuss the generalisability (external validity) of the study results DONE – see lines 463-594 |
| Other information | | |
| Funding | 22 | Give the source of funding and the role of the funders for the present study and, if applicable, for the original study on which the present article is based DONE – information given on online system |

*Give information separately for cases and controls in case-control studies and, if applicable, for exposed and unexposed groups in cohort and cross-sectional studies.

**Note:** An Explanation and Elaboration article discusses each checklist item and gives methodological background and published examples of transparent reporting. The STROBE checklist is best used in conjunction with this article (freely available on the Web sites of PLoS Medicine at http://www.plosmedicine.org/, Annals of Internal Medicine at http://www.annals.org/, and Epidemiology at http://www.epidem.com/). Information on the STROBE Initiative is available at www.strobe-statement.org.
